# A single next generation sequencing assay for detection of driver mutations, rearrangements and copy number abnormalities in plasma cell dyscrasias

**DOI:** 10.1038/s41408-026-01482-2

**Published:** 2026-03-28

**Authors:** Cecilia Bonolo de Campos, Daniela Trujillo, James Smadbeck, Mariano Arribas, Hongwei Tang, Neeraj Sharma, Gregory J. Ahmann, Shaji K. Kumar, A. Keith Stewart, Rafael Fonseca, P. Leif Bergsagel, Yan W. Asmann, Linda B. Baughn, Esteban Braggio

**Affiliations:** 1 Princess Margaret Cancer Center, University Health Network, Toronto, ON Canada; 2 Division of Hematology and Oncology, Mayo Clinic, Phoenix, AZ USA; 3 Center for Individualized Medicine-Biomarker Discovery, Mayo Clinic, Rochester, MN USA; 4 Department of Laboratory Medicine and Pathology, Division of Hematopathology, Mayo Clinic, Rochester, MN USA; 5 Department of Internal Medicine, Division of Hematology, Mayo Clinic, Rochester, MN USA; 6 Department of Quantitative Health Sciences, Mayo Clinic, Jacksonville, FL USA

**Keywords:** Myeloma, Cancer genetics

Multiple myeloma (MM) is a plasma cell malignancy characterized by recurring primary and secondary genetic abnormalities that impact clinical outcome and response to therapy. The International Myeloma Society/International Myeloma Working Group (IMWG/IMS) proposed the Consensus Genomic Staging of high-risk MM, which integrates cytogenetic Fluorescence in situ hybridization (FISH) testing [del(17p), t(4;14), t(14;16), t(14;20), del(1p), and 1q gain or amplification (1q+)], genomic assessment of *TP53* mutational status, and biomarker evaluation (b2-microglobulin and creatinine levels) [1]. The increasing importance of genomic characterization of MM was further explored with the risk-prediction model for newly diagnosed MM, combining clinical, treatment, and 20 genomic variables, including 1q+, del(1p), *TP53* loss, *NSD2* translocations, APOBEC mutational signatures, and copy-number signatures [2].

FISH remains the current gold standard assay to detect clinically relevant copy-number abnormalities (CNA) and structural variants (SV). This method, however, has limitations in the real-world clinical laboratory setting, including the restricted detection of subclonal copy number abnormalities and the interrogation of a limited number of chromosomal regions, frequently excluding rare, but clinically relevant, abnormalities, such as translocations of the immunoglobulin light chain, and incomplete detection of *MYC* rearrangements [3, 4]. Furthermore, FISH is unable to detect mutations such as single-nucleotide variants and small insertions and deletions, including those involving *TP53* [5, 6], a key feature of high-risk MM defined by the IMWG/IMS [1]. In this study, we present a targeted sequencing single-assay approach that simultaneously detects SV, CNA, and clinically relevant mutations in MM.

Primary patient-derived CD138+ cells were enriched from bone marrow aspirates using magnetic beads. Clinical and laboratory annotations were abstracted, including FISH reports and clinical follow-up. Written informed consent was obtained, and samples were collected and stored under Mayo Clinic’s Institutional Review Board approval (IRBs 919-04 and 521-93). The study was conducted in accordance with the Declaration of Helsinki.

DNA was isolated using the AllPrep DNA/RNA Kit according to the manufacturers’ instructions. The entire coding regions of 139 genes, regions surrounding *MYC*, immunoglobulin heavy chain (IgH), and immunoglobulin light chain kappa (IgK) and lambda (IgL) loci and additional probes distributed across the genome for copy number estimation were sequenced using a customized 2.3 Mb SureSelect gene panel (Supplemental Table 1). Samples were paired-end sequenced (150 bp reads) in an Illumina HiSeq 4000. Raw variant quality was annotated using the GATK variant annotator [7], somatic mutations were called with MuTect2 in tumor-only mode [8], and variant annotation was performed using Biological Reference Repository (BioR) [9], including variant deleteriousness prediction.

Detailed methods on the DNA sequencing analyzes performed, including SNV, SVs, and CNAs can be found in the Supplemental Methods. The R packages “survival” (version-3.8-3) and “survminer” (version-0.5.0) were used to visualize and compare (log-rank test) overall survival curves of patients with newly diagnosed MM with high- and low-risk genetics determined by FISH testing or sequencing based on the IMWG/IMS 2025 risk stratification guidelines.

The present study included 264 primary patient samples. Most (95%) samples were obtained from individuals diagnosed with MM, 3% as smoldering MM (SMM) and 2% as monoclonal gammopathy of undetermined significance (MGUS). The MM samples were further subdivided into 81% of newly diagnosed cases, while 19% of MM samples were collected at relapse (mean number of previous treatment regimens = 4.2, range 1–14).

We identified a median of two mutated genes per sample (range 0–13) (Supplemental Table 2). The most frequently mutated genes were: *KRAS* (34%), *NRAS* (25%), *FAM46C* (15%), *DIS3* (14%), *BRAF* (13%), *ATM* (12%), and *TP53* (11%) (Fig. 1).

**Fig. 1:**
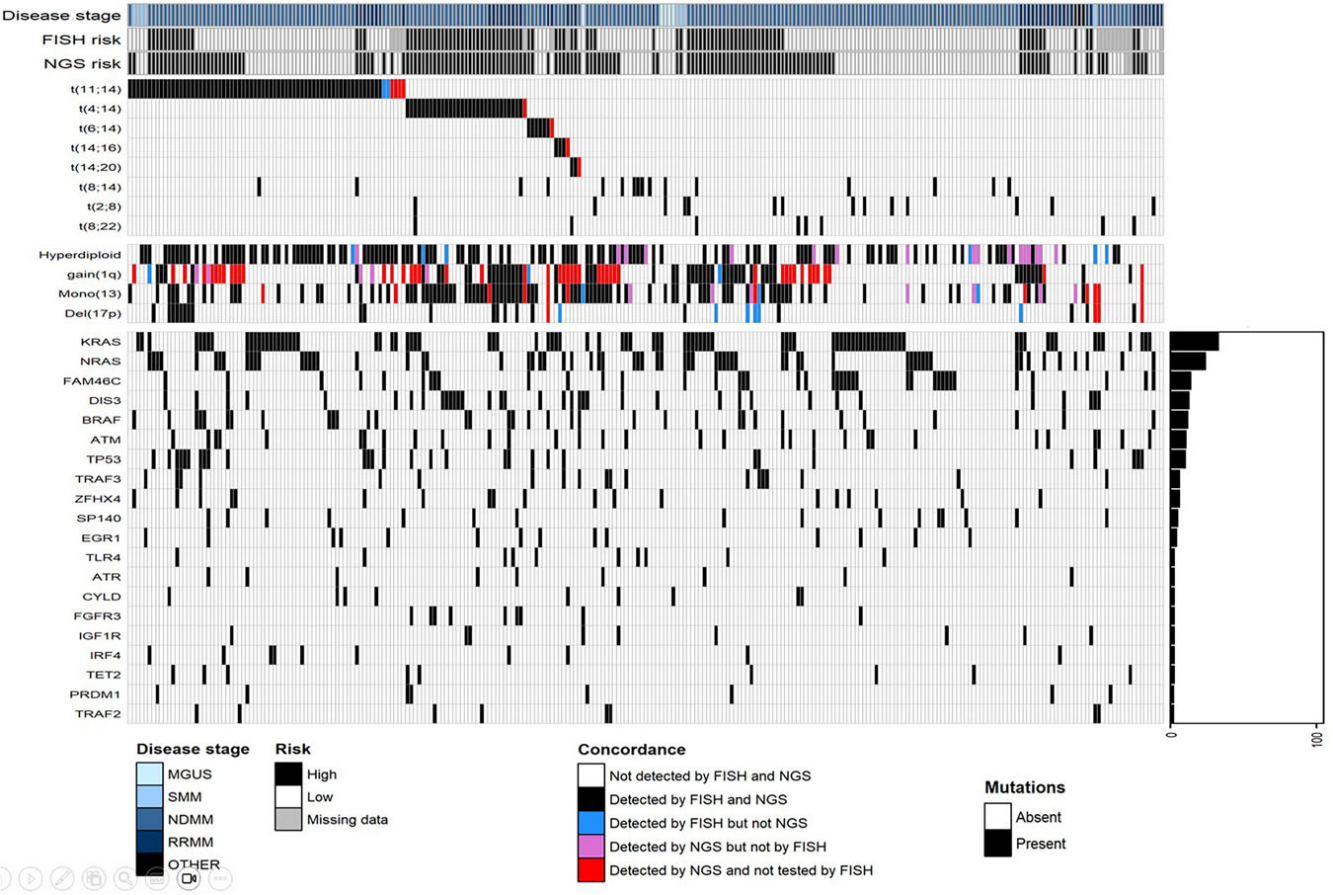
Summary of CNV, SV and mutations found in the cohort. Concordance between FISH and NGS in the detection of rearrangements and copy number variations (CNV), in addition to the driver mutation profile detected by NGS, risk stratification determined by FISH and NGS and the diagnosis of all 264 primary patient samples.

FISH was unavailable for 18 (6.8%) cases. The remaining samples were assessed with the complete MM FISH panel at diagnosis [dual-fusion probes for t(4;14), t(11;14), t(14;16), t(14;20), t(6;14), breakapart probe for IGH, trisomy of chromosomes 3, 7, 9, 11, and 15, del(17p), 1q+, del(13q) and *MYC* rearrangements], and 21 cases (8%) had an additional sample collected and assessed with the progression MM FISH panel, specifically evaluating for del (17), 1q+, and *MYC* rearrangements. The level of detection required to identify the abnormalities by FISH included 6% for dual-fusion probes [e.g., t(11;14)], 10% for disrupted or separated probes (i.e., IGH) and 20% for enumeration probes (i.e., *TP53*).

Comparing the sequencing panel with FISH, there was 100% sensitivity and specificity for the identification of t(4;14)(IGH::*FGFR3* or IGH::*NSD2)*, t(6;14)(IGH::*CCND3)*, t(14;16)(IGH::*MAF)*, and t(14;20)(IGH::*MAFB)*; and a 100% specificity and 97% sensitivity for t(11;14)(IGH::*CCND1)* (Supplemental Table 3). We also identified a rare case of a double IGH rearrangement, t(11;14) and t(14;16), similar to previous reports [10].

Out of 19 samples with an abnormal IGH rearrangement without a known partner by FISH, our panel identified an t(8;14)(IGH::*MYC*) in 6 cases (31.5%). Of note, those cases were originally analyzed before implementing the study of *MYC* alterations in the clinical laboratory. Furthermore, we identified *MYC* rearrangements with the immunoglobulin light chain genes in 22 cases: 14 cases with t(2;8)(IGK::*MYC*), and eight cases with t(8;22)(IGL::*MYC*). Although IGL rearrangements, particularly IGL::*MYC* rearrangements, have been associated with poor prognosis, they are not routinely evaluated in FISH assays [11].

We identified 142 cases with hyperdiploid MM (H-MM). Of these, 119 (83.8%) were detected by both FISH and sequencing, 6 (4.2%) were detected only by FISH. Furthermore, in 17 (11.9%) cases with an incomplete FISH panel, we were able to detect H-MM by sequencing. When comparing methods for the detection of H-MM, we found a sensitivity of 95%, specificity of 85%, PPV of 87% and NPV of 95% using the sequencing approach.

The sequencing panel was also able to detect gain or amplification of 1q with 95% sensitivity and 91% specificity, and monosomy 13 or del(13q) with 97% sensitivity and 95% specificity. Del(17p) was initially detected with 59% sensitivity and 100% specificity. The reduced sensitivity for del(17p) detection was mostly due to the presence of subclonal events. Because *TP53* is included in the custom capture panel, we increased the sensitivity by determining six separate regions of coverage using on-target probes, which enabled the detection of focal del(17p), which accounted for most of the false negative cases (54%), increasing the sensitivity to 78% and the NPV to 97.5%.

Of 27 samples with del(17p) by FISH, we identified *TP53* mutations (i.e., biallelic inactivation) in 14 samples (45%). Furthermore, we detected an additional 17 cases with *TP53* mutations and no del(17p) by FISH, four of which had *TP53* double-hit mutations and/or *TP53* mutations with Variant Allele Frequency (VAF) > 50%, suggesting the presence of copy neutral loss of heterozygosity (CN-LOH) (Supplemental Table 4).

Lastly, the risk stratification and clinical utility of the sequencing assay was demonstrated by the shorter overall survival observed in all samples (MGUS, SMM, NDMM and RRMM) classified as high risk (hazard ratio [HR] = 3.66; CI:2.39–5.59; *p* < 0.0001), which was also observed with risk assessment by FISH (HR = 4.67; CI:2.45–8.91; *p* < 0.0001; Fig. 2).

**Fig. 2:**
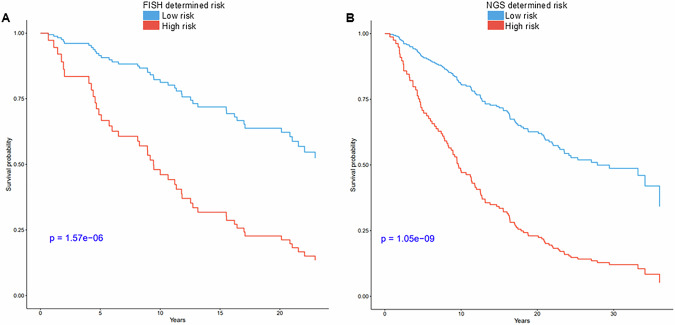
Kaplan–Meier survival curve by risk stratification. **A** Overall survival between high (*N* = 95) and low risk (*N* = 151) determined by FISH. **B** Overall survival between high (*N* = 144) and low risk (*N* = 117) determined by NGS.

Multiple myeloma is characterized by a wide variety of genomic abnormalities, including copy number gains and losses, immunoglobulin gene rearrangements, and genetic mutations, all of which have a significant impact on disease outcome and prognosis [12]. While FISH remains the current gold standard assay, the heterogeneity of the disease could benefit from additional tools for comprehensive genetic characterization. The advent of sequencing has enhanced our ability to detect mutations that are now required for MM risk stratification [13].

Here, we found high concordance for rearrangements, CNAs and ploidy between FISH and the sequencing panel. However, 13 cases of del(17p) were not detected by NGS, but were detected by FISH, highlighting a limitation of targeted sequencing for the detection of del(17p). Since some of these false negative cases were due to low tumor cell content, ensuring a tumor purity >20% should reduce the risk of these false negative findings or supplementation of the NGS panel test with FISH for del(17p) might be recommended.

In cases with del(17p), sequencing enables the identification of additional cases with single hit (mutation only), LOH or biallelic hits, which may confer a worse prognosis than del(17p) alone [14]. All these different scenarios in this population of *TP53*-abnormal cases demonstrate the importance of accurately categorizing patients into distinct risk groups, supporting the idea of incorporating additional laboratory techniques that provide deeper genetic insights [14].

While FISH remains the gold standard for MM due to its established clinical value, multiple novel genomic approaches are emerging. Compared with targeted sequencing, whole genome sequencing is a powerful approach for a comprehensive genomic characterization, but its use in the clinical diagnostic labs is still restricted, mostly due to the higher cost, the computationally demanding bioinformatic analyzes of large datasets and the moderate coverage depth, limiting the detection of low variant allelic frequency mutations [15]. Therefore, targeted sequencing is a more cost-efficient choice in detecting clinically relevant abnormalities in MM. The detection of relevant abnormalities not routinely assessed in most clinical labs, including *TP53* mutations, IGL and *MYC* rearrangements, supports the use of this small, targeted panel as a clinically relevant, single molecular assay for the comprehensive profiling of MM. Some limitations remained, including the partial detection of subclonal del(17p) events, and potentially the t(11;14), which could be supplemented by a FISH analysis. The complementary strengths of both methods enable a more accurate and clinically meaningful assessment of MM.

## Supplementary information

Supplemental material

Checklist

## Data Availability

All data generated or analysed during this study are included in this published article and its supplementary information files. Raw data is available upon request.
