# Supplementary material for: A single next generation sequencing assay for detection of driver mutations, rearrangements and copy number abnormalities in plasma cell dyscrasias
Source: Blood Cancer J. 2026 Mar 28;16(1):45. doi: 10.1038/s41408-026-01482-2 (PMC13032923; doi:10.1038/s41408-026-01482-2)
Supplement: Supplementary file 1 — Supplemental material [file 41408_2026_1482_MOESM1_ESM.docx]

**A single next generation sequencing assay for detection of driver mutations, rearrangements and copy number abnormalities in plasma cell dyscrasias**

**Supplementary Information Summary**This supplementary file contains detailed sequencing methods, the full gene list in the custom SureSelect panel, the complete catalog of somatic variants identified in all patients, and the diagnostic performance metrics (sensitivity, specificity, PPV, and NPV) for rearrangements and copy number variations detected by FISH and NGS.

**Supplemental Methods**

DNA was isolated using the AllPrep DNA/RNA Kit according to the manufacturers’ instructions. The entire coding regions of 139 genes, regions surrounding MYC, immunoglobulin heavy chain (IgH), and immunoglobulin light chain kappa (IgK) and lambda (IgL) loci and additional probes distributed across the genome for copy number estimation were sequenced using a customized 2.3 Mb SureSelect gene panel (Supplemental Table 1). Samples were paired end sequenced (150 bp reads) in an Illumina HiSeq 4000. Raw variant quality was annotated using the GATK variant annotator [7], somatic mutations were called with MuTect2 in tumor-only mode[8], and variant annotation was performed using Biological Reference Repository (BioR)[9], including variant deleteriousness prediction. Germline mutations were eliminated when minor allele frequencies (>0.01%) were identified in germline variant databases (gnomAD, 1000 Genomes Project, ExAC, and ESP6500); except for mutations involving known MM mutation hotspots or found in COSMIC. Additionally, all variants with less than 10 supportive reads or <1% VAF were eliminated.

Chromosomal rearrangements were identified using an in-house developed algorithm designed specifically for targeted capture DNA sequencing data analyses and for resolving the reads mapping and SV calling in the complex Immunoglobulin regions. SVs were called based on discordant read pairs as well as junction split reads, solving the challenges from the overlapping paired end reads due to short DNA fragments and the mapping ambiguities in the Ig regions. False SVs were filtered according to the following criteria: (I) the number of the supporting read pairs and junction split reads; (II) the lack of SV calls at the same regionsfrom in-house non-MM control datasets and public SV germline databases; (III) the mappability and uniqueness of the region based on data from the ENCODE project; (IV) consistencies of the mapping orientations of the supporting read pairs.

CNAs were assessed using both on and off target reads, and a 200kb bin size for segmentation covering the entire genome. Off-target coverage was assessed by filtering the aligned reads for the inverse of the targeted panel’s footprint in .bed format. The coverage was calculated as the number of reads aligned to each 200kb region across the genome. GC correction was performed by first splitting the coverage data into GC content bins of quantiles ranging from 1% to 99% with steps of 1%. Median coverage was taken for each GC content bin and a linear function fit to the data for GC correction. Segmentation was performed using a 1Mb sliding window approach. Adjacent windows that deviated >3 standard deviations in coverage constituted boundaries of copy number segments. These segments were then assessed against the expected coverage value for 2N diploid, which was taken as the mode of coverage across the genome. Segments calculated as 2.2N in normalized coverage were called “gain”. For aneuploidy assessment, if the majority (>50%) of an arm or chromosome was calculated as “gain” or “loss” then the whole arm or chromosome was called “gain” or “loss”, respectively.

**Supplemental Tables**

**Supplementary Table 1: SureSelect gene panel.**

| ACTG1 | EGFR | NR3C1 | PSMC2 | RASA2 |
| --- | --- | --- | --- | --- |
| AKT1 | EGR1 | NRAS | PSMC3 | RB1 |
| AKT2 | EIF2AK3 | PIK3CA | PSMC3IP | RIPK1 |
| AKT3 | ERN1 | PIK3CG | PSMC4 | RIPK4 |
| ATF4 | FAM46C | PIK3R1 | PSMC5 | SHC1 |
| ATF6 | FGFR3 | PIK3R2 | PSMC6 | SP140 |
| ATM | GRB2 | PIM1 | PSMD1 | STAT3 |
| ATR | HSPA5 | PIM2 | PSMD10 | TET2 |
| B2M | IDH1 | PIM3 | PSMD11 | TGFBR2 |
| BIRC2 | IDH2 | PRDM1 | PSMD12 | TLR4 |
| BIRC3 | IDH3A | PSMA1 | PSMD13 | TNFRSF13B |
| BRAF | IFNGR2 | PSMA2 | PSMD14 | TNFRSF21 |
| BTG1 | IGF1R | PSMA3 | PSMD2 | TP53 |
| CARD11 | IKZF1 | PSMA4 | PSMD3 | TRAF2 |
| CCND1 | IKZF3 | PSMA5 | PSMD4 | TRAF3 |
| CCNT1 | IL6 | PSMA6 | PSMD5 | TRAF3IP1 |
| CD38 | IL6R | PSMA7 | PSMD6 | WHSC1 |
| CDK4 | IL6ST | PSMA8 | PSMD7 | XBP1 |
| CDK7 | IRF4 | PSMB1 | PSMD8 | ZFHX4 |
| CDKN1B | JAK2 | PSMB10 | PSMD9 |  |
| CDKN2A | KDM6A | PSMB11 | PSME1 |  |
| CDKN2C | KRAS | PSMB2 | PSME2 |  |
| CRBN | MAF | PSMB3 | PSME3 |  |
| CUL4A | MAFB | PSMB4 | PSME4 |  |
| CUL4B | MAX | PSMB5 | PSMF1 |  |
| CXCR4 | MYC | PSMB6 | PSMG1 |  |
| CYLD | MYD88 | PSMB7 | PSMG2 |  |
| DDIT3 | NFKB2 | PSMB8 | PSMG3 |  |
| DIS3 | NFKBIA | PSMB9 | PSMG4 |  |
| DUSP2 | NFKBIB | PSMC1 | PTPN11 |  |

**Supplementary table 2: List of mutations detected by the targeted next-generation sequencing (NGS) assay in patients with plasma cell dyscrasias. For each variant, the sample ID, genomic coordinates (CHROM:POS), reference (REF) and alternative (ALT) alleles, variant allele frequency (VAF), transcript ID (CAVA_TRANSCRIPT), gene name (CAVA_GENE), and predicted sequence ontology (CAVA_SO) are shown.**

| **Sample ID** | **CHROM:POS** | **REF** | **ALT** | **VAF** | **CAVA_TRANSCRIPT** | **CAVA_GENE** | **CAVA_SO** |
| --- | --- | --- | --- | --- | --- | --- | --- |
| **MM1** | chr8:77619936 | A | C | 0.026 | ENST00000521891 | ZFHX4 | missense_variant |
| **MM2** | chr7:140453136 | A | T | 0.389 | ENST00000288602 | BRAF | missense_variant |
| **MM2** | chr2:231118030 | G | A | 0.515 | ENST00000392045 | SP140 | splice_acceptor_variant |
| **MM3** | chr2:136872912 | C | T | 0.458 | ENST00000241393 | CXCR4 | missense_variant |
| **MM3** | chr12:25380275 | T | G | 0.212 | ENST00000311936 | KRAS | missense_variant |
| **MM3** | chr14:103371630 | T | TTCCAG | 0.049 | ENST00000560371 | TRAF3 | frameshift_variant |
| **MM3** | chr4:106164892 | A | G | 0.5 | ENST00000380013 | TET2 | missense_variant |
| **MM4** | chr3:30713834 | G | A | 0.606 | ENST00000295754 | TGFBR2 | missense_variant |
| **MM4** | chr11:47442252 | C | A | 0.321 | ENST00000298852 | PSMC3 | missense_variant |
| **MM4** | chr13:73335853 | A | T | 0.405 | ENST00000377767 | DIS3 | stop_gained |
| **MM5** | chr6:47253928 | C | T | 0.536 | ENST00000296861 | TNFRSF21 | missense_variant |
| **MM6** |  |  |  |  |  |  |  |
| **MM7** | chr12:25380275 | T | G | 0.379 | ENST00000311936 | KRAS | missense_variant |
| **MM8** | chr9:120475431 | T | A | 0.48 | ENST00000355622 | TLR4 | missense_variant |
| **MM8** | chr11:108199845 | C | G | 0.341 | ENST00000278616 | ATM | missense_variant |
| **MM8** | chr12:25380275 | T | G | 0.417 | ENST00000311936 | KRAS | missense_variant |
| **MM9** | chr4:1803568 | C | G | 0.133 | ENST00000440486 | FGFR3 | missense_variant |
| **MM9** | chr13:73335929 | C | T | 0.031 | ENST00000377767 | DIS3 | missense_variant |
| **MM9** | chr13:73345958 | C | T | 0.144 | ENST00000377767 | DIS3 | missense_variant |
| **MM9** | chr13:73355129 | C | T | 0.351 | ENST00000377767 | DIS3 | missense_variant |
| **MM9** | chr17:7577566 | T | C | 0.281 | ENST00000269305 | TP53 | missense_variant |
| **MM10** | chr12:25378647 | T | G | 0.032 | ENST00000311936 | KRAS | missense_variant |
| **MM10** | chr7:140453136 | A | T | 0.048 | ENST00000288602 | BRAF | missense_variant |
| **MM10** | chr11:108236195 | A | G | 0.013 | ENST00000278616 | ATM | missense_variant |
| **MM10** | chr7:55219023 | G | T | 0.147 | ENST00000275493 | EGFR | missense_variant |
| **MM10** | chr1:115256529 | T | C | 0.028 | ENST00000369535 | NRAS | missense_variant |
| **MM10** | chr17:30791070 | G | A | 0.041 | ENST00000261712 | PSMD11 | missense_variant |
| **MM11** | chr12:25378561 | G | A | 0.012 | ENST00000311936 | KRAS | missense_variant |
| **MM11** | chr12:25378647 | T | A | 0.015 | ENST00000311936 | KRAS | missense_variant |
| **MM11** | chr12:25398284 | C | A | 0.022 | ENST00000311936 | KRAS | missense_variant |
| **MM12** | chr14:35872526 | ATTTC | A | 0.13 | ENST00000216797 | NFKBIA | frameshift_variant |
| **MM12** | chr14:103363706 | C | T | 0.792 | ENST00000560371 | TRAF3 | stop_gained |
| **MM12** | chr14:103371884 | T | G | 0.063 | ENST00000560371 | TRAF3 | missense_variant |

| **MM12** | chr17:7577568 | C |  | A | 0.606 | ENST00000269305 | TP53 | missense_variant |
| --- | --- | --- | --- | --- | --- | --- | --- | --- |
| **MM13** | chr15:90631596 | C |  | T | 0.392 | ENST00000330062 | IDH2 | missense_variant |
| **MM13** | chr6:106547242 | G |  | A | 0.43 | ENST00000369096 | PRDM1 | missense_variant |
| **MM14** | chr1:152187116 | C |  | T | 0.063 | ENST00000368801 | HRNR | missense_variant |
| **MM14** | chr1:115256529 | T |  | C | 0.019 | ENST00000369535 | NRAS | missense_variant |
| **MM14** | chr12:25378647 | T |  | A | 0.038 | ENST00000311936 | KRAS | missense_variant |
| **MM14** | chr13:73336064 | C |  | G | 0.162 | ENST00000377767 | DIS3 | missense_variant |
| **MM14** | chr5:142779588 | GTGTTACAT |  | G | 0.115 | ENST00000343796 | NR3C1 | frameshift_variant |
| **MM15** | chr8:77768003 | G |  | C | 0.451 | ENST00000521891 | ZFHX4 | missense_variant |
| **MM16** | chr8:77617292 | G |  | C | 0.486 | ENST00000521891 | ZFHX4 | missense_variant |
| **MM16** | chr7:50459525 | G |  | A | 0.014 | ENST00000331340 | IKZF1 | missense_variant |
| **MM16** | chr3:30713834 | G |  | T | 0.466 | ENST00000295754 | TGFBR2 | missense_variant |
| **MM16** | chr1:243716046 | T |  | A | 0.209 | ENST00000263826 | AKT3 | missense_variant |
| **MM16** | chr14:103371556 | TG |  | T | 0.326 | ENST00000560371 | TRAF3 | frameshift_variant |
| **MM16** | chr14:103371672 | TGGAAGATTCG |  | T | 0.531 | ENST00000560371 | TRAF3 | frameshift_variant |
| **MM16** | chr17:7578203 | C |  | T | 0.354 | ENST00000269305 | TP53 | missense_variant |
| **MM16** | chr9:120474680 | A |  | G | 0.486 | ENST00000355622 | TLR4 | missense_variant |
| **MM17** | chr18:12720543 | C |  | A | 0.408 | ENST00000317615 | PSMG2 | missense_variant |
| **MM17** | chr1:115256529 | T |  | C | 0.444 | ENST00000369535 | NRAS | missense_variant |
| **MM18** | chr12:122337662 | C |  | T | 0.415 | ENST00000261817 | PSMD9 | missense_variant |
| **MM18** | chr1:118166408 | C |  | G | 0.359 | ENST00000369448 | FAM46C | stop_gained |
| **MM18** | chr2:231134587 | TC |  | T | 0.402 | ENST00000392045 | SP140 | frameshift_variant |
| **MM19** | chr4:106157419 | T |  | C | 0.645 | ENST00000380013 | TET2 | missense_variant |
| **MM20** | chr4:106197606 | C |  | T | 0.486 | ENST00000380013 | TET2 | missense_variant |
| **MM20** | chr1:115256529 | T |  | C | 0.221 | ENST00000369535 | NRAS | missense_variant |
| **MM20** | chr1:115256530 | G |  | T | 0.015 | ENST00000369535 | NRAS | missense_variant |
| **MM20** | chr9:120476472 | G |  | A | 0.036 | ENST00000355622 | TLR4 | missense_variant |
| **MM20** | chr9:128000943 | C |  | T | 0.011 | ENST00000324460 | HSPA5 | missense_variant |
| **MM21** | chr1:118166361 | T |  | TA | 0.932 | ENST00000369448 | FAM46C | frameshift_variant |
| **MM21** | chr12:25398281 | C |  | T | 0.158 | ENST00000311936 | KRAS | missense_variant |
| **MM21** | chr7:140481411 | C |  | A | 0.068 | ENST00000288602 | BRAF | missense_variant |
| **MM22** | chr1:115256529 | T |  | C | 0.024 | ENST00000369535 | NRAS | missense_variant |
| **MM22** | chr1:152187105 | C |  | T | 0.066 | ENST00000368801 | HRNR | missense_variant |
| **MM22** | chr12:25398281 | C |  | T | 0.112 | ENST00000311936 | KRAS | missense_variant |

| **MM22** | chr12:25398284 | C | T | 0.2 | ENST00000311936 | KRAS | missense_variant |
| --- | --- | --- | --- | --- | --- | --- | --- |
| **MM22** | chr8:77767132 | C | T | 0.017 | ENST00000521891 | ZFHX4 | missense_variant |
| **MM23** | chr16:74339360 | A | G | 0.5 | ENST00000219313 | PSMD7 | missense_variant |
| **MM23** | chr10:104157424 | C | T | 0.067 | ENST00000369966 | NFKB2 | stop_gained |
| **MM23** | chr10:104158022 | G | T | 0.078 | ENST00000369966 | NFKB2 | missense_variant |
| **MM23** | chr10:104160747 | C | A | 0.057 | ENST00000369966 | NFKB2 | missense_variant |
| **MM23** | chr10:104161005 | G | A | 0.413 | ENST00000369966 | NFKB2 | missense_variant |
| **MM23** | chr12:25380264 | C | A | 0.452 | ENST00000311936 | KRAS | missense_variant |
| **MM23** | chr14:53178135 | C | T | 0.025 | ENST00000606149 | PSMC6 | missense_variant |
| **MM24** | chr1:115258744 | C | T | 0.02 | ENST00000369535 | NRAS | missense_variant |
| **MM24** | chr1:115258747 | C | T | 0.325 | ENST00000369535 | NRAS | missense_variant |
| **MM24** | chr1:152187183 | C | A | 0.517 | ENST00000368801 | HRNR | missense_variant |
| **MM24** | chr8:7673126 | C | A | 0.529 | ENST00000335021 | DEFB107A | missense_variant |
| **MM24** | chr1:143767513 | A | T | 0.306 | ENST00000419275 | PPIAL4G | missense_variant |
| **MM24** | chr1:144865850 | G | T | 0.52 | ENST00000369356 | PDE4DIP | missense_variant |
| **MM24** | chr7:140481403 | C | T | 0.264 | ENST00000288602 | BRAF | missense_variant |
| **MM25** | chr3:178938838 | G | T | 0.474 | ENST00000263967 | PIK3CA | missense_variant |
| **MM25** | chr11:69456220 | T | A | 0.507 | ENST00000227507 | CCND1 | missense_variant |
| **MM25** | chr11:69456265 | A | T | 0.465 | ENST00000227507 | CCND1 | missense_variant |
| **MM25** | chr12:25378643 | C | G | 0.029 | ENST00000311936 | KRAS | missense_variant |
| **MM25** | chr14:103342746 | G | A | 0.018 | ENST00000560371 | TRAF3 | missense_variant |
| **MM25** | chr17:7577085 | C | T | 0.014 | ENST00000269305 | TP53 | missense_variant |
| **MM25** | chr17:40986589 | C | G | 0.027 | ENST00000293362 | PSME3 | missense_variant |
| **MM25** | chr2:54102817 | G | A | 0.063 | ENST00000404125 | PSME4 | stop_gained |
| **MM25** | chr22:39918119 | G | A | 0.022 | ENST00000337304 | ATF4 | missense_variant |
| **MM25** | chr3:63996373 | G | A | 0.029 | ENST00000492933 | PSMD6 | stop_gained |
| **MM25** | chr3:141290261 | C | A | 0.148 | ENST00000452898 | RASA2 | stop_gained |
| **MM25** | chr3:141295841 | G | C | 0.02 | ENST00000452898 | RASA2 | splice_acceptor_variant |
| **MM25** | chr7:140453155 | C | T | 0.013 | ENST00000288602 | BRAF | missense_variant |
| **MM25** | chr8:77618763 | G | A | 0.015 | ENST00000521891 | ZFHX4 | missense_variant |
| **MM25** | chr8:128752949 | G | C | 0.056 | ENST00000377970 | MYC | missense_variant |
| **MM25** | chrX:119691895 | C | G | 0.053 | ENST00000404115 | CUL4B | splice_acceptor_variant |
| **MM26** | chr5:67569315 | C | A | 0.511 | ENST00000521381 | PIK3R1 | splice_donor_5th_base_varian t |
| **MM26** | chr16:50813764 | C | T | 0.102 | ENST00000311559 | CYLD | stop_gained |

| **MM26** | chr16:50828150 | C |  | T | 0.483 | ENST00000311559 | CYLD | missense_variant |
| --- | --- | --- | --- | --- | --- | --- | --- | --- |
| **MM26** | chr17:7577563 | T |  | C | 0.2 | ENST00000269305 | TP53 | missense_variant |
| **MM26** | chr7:140453155 | C |  | T | 0.018 | ENST00000288602 | BRAF | missense_variant |
| **MM27** | chr1:118165865 | AGTCACTCTGA |  | C | 0.035 | ENST00000369448 | FAM46C | inframe_deletion |
| **MM27** | chr1:118165871 | CAA |  | C | 0.18 | ENST00000369448 | FAM46C | frameshift_variant |
| **MM27** | chr1:118166146 | T |  | TTGA | 0.054 | ENST00000369448 | FAM46C | inframe_insertion |
| **MM27** | chr1:118166221 | T |  | C | 0.011 | ENST00000369448 | FAM46C | missense_variant |
| **MM27** | chr1:118166357 | GACTT |  | G | 0.028 | ENST00000369448 | FAM46C | frameshift_variant |
| **MM27** | chr1:118166386 | AAG |  | A | 0.082 | ENST00000369448 | FAM46C | frameshift_variant |
| **MM27** | chr12:25398281 | C |  | T | 0.42 | ENST00000311936 | KRAS | missense_variant |
| **MM27** | chr13:73335857 | A |  | G | 0.533 | ENST00000377767 | DIS3 | missense_variant |
| **MM27** | chr5:68555711 | G |  | A | 0.024 | ENST00000256443 | CDK7 | missense_variant |
| **MM28** | chr12:25398284 | C |  | T | 0.207 | ENST00000311936 | KRAS | missense_variant |
| **MM28** | chr11:108158409 | C |  | T | 0.015 | ENST00000278616 | ATM | missense_variant |
| **MM28** | chr12:25380268 | A |  | C | 0.068 | ENST00000311936 | KRAS | missense_variant |
| **MM28** | chr14:103371798 | G |  | T | 0.788 | ENST00000560371 | TRAF3 | missense_variant |
| **MM29** | chr11:102206862 | A |  | G | 0.266 | ENST00000263464 | BIRC3 | missense_variant |
| **MM29** | chr1:151239008 | C |  | T | 0.458 | ENST00000368884 | PSMD4 | missense_variant |
| **MM29** | chr3:3215870 | G |  | A | 0.421 | ENST00000231948 | CRBN | missense_variant |
| **MM29** | chr1:152187111 | C |  | T | 0.267 | ENST00000368801 | HRNR | missense_variant |
| **MM30** | chr7:140481402 | C |  | T | 0.015 | ENST00000288602 | BRAF | missense_variant |
| **MM30** | chr11:108143465 | GGCCATTCTTA |  | A | 0.092 | ENST00000278616 | ATM | inframe_deletion |
| **MM30** | chr13:73355017 | T |  | G | 0.825 | ENST00000377767 | DIS3 | missense_variant |
| **MM30** | chr14:103371729 | TC |  | T | 0.036 | ENST00000560371 | TRAF3 | frameshift_variant |
| **MM30** | chr14:103371884 | T |  | G | 0.088 | ENST00000560371 | TRAF3 | missense_variant |
| **MM30** | chr2:96810559 | G |  | C | 0.516 | ENST00000288943 | DUSP2 | missense_variant |
| **MM30** | chr2:96810607 | AG |  | A | 0.304 | ENST00000288943 | DUSP2 | frameshift_variant |
| **MM31** | chr13:73336078 | A |  | T | 0.962 | ENST00000377767 | DIS3 | missense_variant |
| **MM30** | chr4:1803171 | CCGCTGGCAA |  | C | 0.154 | ENST00000440486 | FGFR3 | inframe_deletion |
| **MM30** | chr4:1803640 | TCCACTG |  | T | 0.229 | ENST00000440486 | FGFR3 | inframe_deletion |
| **MM32** | chr12:25380275 | T |  | G | 0.04 | ENST00000311936 | KRAS | missense_variant |
| **MM32** | chr13:73346389 | T |  | A | 0.733 | ENST00000377767 | DIS3 | missense_variant |
| **MM32** | chr17:7577547 | C |  | T | 0.017 | ENST00000269305 | TP53 | missense_variant |
| **MM32** | chr4:1807889 | A |  | G | 0.788 | ENST00000440486 | FGFR3 | missense_variant |

| **MM32** | chr6:106554951 | TCCCAGTGC | T | 0.071 | ENST00000369096 | PRDM1 | frameshift_variant |
| --- | --- | --- | --- | --- | --- | --- | --- |
| **MM33** | chr15:99456467 | G | A | 0.561 | ENST00000268035 | IGF1R | missense_variant |
| **MM33** | chr1:154408560 | C | T | 0.386 | ENST00000368485 | IL6R | missense_variant |
| **MM33** | chr14:103341969 | GGATAATT | G | 0.485 | ENST00000560371 | TRAF3 | frameshift_variant |
| **MM33** | chr17:79479364 | G | A | 0.371 | ENST00000331925 | ACTG1 | missense_variant |
| **MM33** | chr5:137801453 | G | A | 0.53 | ENST00000239938 | EGR1 | initiator_codon_variant |
| **MM33** | chr9:139820153 | G | T | 0.054 | ENST00000247668 | TRAF2 | missense_variant |
| **MM34** | chr1:115256529 | T | A | 0.49 | ENST00000369535 | NRAS | missense_variant |
| **MM34** | chr1:115256530 | G | T | 0.49 | ENST00000369535 | NRAS | missense_variant |
| **MM34** | chr6:394972 | A | G | 0.419 | ENST00000380956 | IRF4 | missense_variant |
| **MM35** | chr1:115256530 | G | T | 0.012 | ENST00000369535 | NRAS | missense_variant |
| **MM35** | chr12:25380275 | T | A | 0.461 | ENST00000311936 | KRAS | missense_variant |
| **MM35** | chr13:73336064 | C | T | 0.426 | ENST00000377767 | DIS3 | missense_variant |
| **MM35** | chr3:142222284 | A | AAT | 0.045 | ENST00000350721 | ATR | frameshift_variant |
| **MM35** | chr6:106553027 | C | T | 0.026 | ENST00000369096 | PRDM1 | missense_variant |
| **MM36** | chr11:102221100 | C | T | 0.498 | ENST00000227758 | BIRC2 | missense_variant |
| **MM36** | chr12:25380276 | T | A | 0.354 | ENST00000311936 | KRAS | missense_variant |
| **MM36** | chr2:239229421 | A | C | 0.455 | ENST00000373327 | TRAF3IP1 | missense_variant |
| **MM37** | chr1:118166331 | A | G | 0.462 | ENST00000369448 | FAM46C | missense_variant |
| **MM37** | chr3:38180483 | C | T | 0.494 | ENST00000417037 | MYD88 | missense_variant |
| **MM37** | chr9:139793344 | GTACTGCTCCT | A | 0.163 | ENST00000247668 | TRAF2 | frameshift_variant |
| **MM37** | chr9:139794901 | G | T | 0.189 | ENST00000247668 | TRAF2 | stop_gained |
| **MM38** | chr11:108224555 | A | G | 0.484 | ENST00000278616 | ATM | missense_variant |
| **MM38** | chr1:152187195 | G | A | 0.059 | ENST00000368801 | HRNR | stop_gained |
| **MM38** | chr12:25378557 | C | A | 0.019 | ENST00000311936 | KRAS | missense_variant |
| **MM39** | chr1:118166018 | G | GT | 0.022 | ENST00000369448 | FAM46C | frameshift_variant |
| **MM39** | chr17:7577022 | G | A | 0.603 | ENST00000269305 | TP53 | stop_gained |
| **MM39** | chr4:1803742 | AAGGTGGGCC | C | 0.079 | ENST00000440486 | FGFR3 | splice_donor_variant |
| **MM39** | chr6:37139177 | A | T | 0.511 | ENST00000373509 | PIM1 | missense_variant |
| **MM40** | chr3:30713665 | G | C | 0.032 | ENST00000295754 | TGFBR2 | missense_variant |
| **MM41** | chr1:118165968 | A | ATG | 0.033 | ENST00000369448 | FAM46C | frameshift_variant |
| **MM41** | chr12:112926872 | C | T | 0.013 | ENST00000351677 | PTPN11 | missense_variant |
| **MM42** | chr2:88887499 | C | T | 0.017 | ENST00000303236 | EIF2AK3 | splice_donor_variant |
| **MM43** | chr11:108186743 | G | A | 0.372 | ENST00000278616 | ATM | missense_variant |

| **MM43** | chr12:25378562 | C | T | 0.025 | ENST00000311936 | KRAS | missense_variant |
| --- | --- | --- | --- | --- | --- | --- | --- |
| **MM43** | chr12:25398281 | C | T | 0.117 | ENST00000311936 | KRAS | missense_variant |
| **MM44** | chr8:77776467 | A | C | 0.465 | ENST00000521891 | ZFHX4 | missense_variant |
| **MM44** | chr7:140453136 | A | T | 0.421 | ENST00000288602 | BRAF | missense_variant |
| **MM44** | chr17:16852066 | G | C | 0.535 | ENST00000261652 | TNFRSF13B | stop_gained |
| **MM44** | chr17:16843694 | A | G | 0.475 | ENST00000261652 | TNFRSF13B | missense_variant |
| **MM44** | chr1:243736228 | C | T | 0.024 | ENST00000263826 | AKT3 | splice_region_variant\| synonymous_variant |
| **MM44** | chr15:99478593 | A | G | 0.52 | ENST00000268035 | IGF1R | missense_variant |
| **MM44** | chr21:43161764 | A | C | 0.506 | ENST00000332512 | RIPK4 | missense_variant |
| **MM44** | chr8:77767306 | A | G | 0.481 | ENST00000521891 | ZFHX4 | missense_variant |
| **MM45** | chr12:58143021 | G | A | 0.5 | ENST00000257904 | CDK4 | missense_variant |
| **MM45** | chr1:118166420 | TCGC | T | 0.469 | ENST00000369448 | FAM46C | inframe_deletion |
| **MM45** | chr15:99460060 | G | A | 0.024 | ENST00000268035 | IGF1R | missense_variant |
| **MM46** | chr9:5064915 | G | T | 0.042 | ENST00000381652 | JAK2 | missense_variant |
| **MM47** | chr1:143767595 | T | C | 0.235 | ENST00000419275 | PPIAL4G | missense_variant |
| **MM47** | chr17:37922121 | CAT | C | 0.015 | ENST00000346872 | IKZF3 | frameshift_variant |
| **MM47** | chr17:61907763 | G | A | 0.112 | ENST00000310144 | PSMC5 | missense_variant |
| **MM47** | chr5:137801694 | AG | A | 0.685 | ENST00000239938 | EGR1 | frameshift_variant |
| **MM47** | chr8:77767898 | G | T | 0.4 | ENST00000521891 | ZFHX4 | missense_variant |
| **MM48** | chr1:51439833 | G | A | 0.383 | ENST00000371761 | CDKN2C | missense_variant |
| **MM48** | chr12:25398284 | C | T | 0.486 | ENST00000311936 | KRAS | missense_variant |
| **MM48** | chr11:69458004 | A | T | 0.692 | ENST00000227507 | CCND1 | missense_variant |
| **MM48** | chr9:120474992 | C | T | 0.01 | ENST00000355622 | TLR4 | missense_variant |
| **MM48** | chr15:78458600 | G | T | 0.481 | ENST00000299518 | IDH3A | missense_variant |
| **MM49** | chr17:37933922 | T | C | 0.026 | ENST00000346872 | IKZF3 | missense_variant |
| **MM49** | chr17:62125256 | A | C | 0.252 | ENST00000433197 | ERN1 | missense_variant |
| **MM50** | chr11:193909 | CTG | C | 0.116 | ENST00000342878 | SCGB1C1 | splice_region_variant\|framesh ift_variant |
| **MM50** | chr12:25380275 | T | G | 0.379 | ENST00000311936 | KRAS | missense_variant |
| **MM51** | chr1:115256529 | T | C | 0.402 | ENST00000369535 | NRAS | missense_variant |
| **MM51** | chr2:231174706 | CAA | C | 0.468 | ENST00000392045 | SP140 | frameshift_variant |
| **MM52** | chr8:77775384 | C | G | 0.457 | ENST00000521891 | ZFHX4 | missense_variant |
| **MM53** | chr12:25398285 | C | A | 0.068 | ENST00000311936 | KRAS | missense_variant |
| **MM53** | chr1:115256530 | G | T | 0.387 | ENST00000369535 | NRAS | missense_variant |
| **MM53** | chr12:12871086 | G | T | 0.72 | ENST00000228872 | CDKN1B | stop_gained |

| **MM53** | chr13:48955381 | AG | A | 0.787 | ENST00000267163 | RB1 | splice_region_variant\|framesh ift_variant |
| --- | --- | --- | --- | --- | --- | --- | --- |
| **MM53** | chr15:78841262 | A | C | 0.435 | ENST00000044462 | PSMA4 | missense_variant |
| **MM53** | chr3:184018144 | C | T | 0.453 | ENST00000310118 | PSMD2 | missense_variant |
| **MM54** | chr17:7574018 | G | A | 0.868 | ENST00000269305 | TP53 | missense_variant |
| **MM54** | chr17:7577130 | A | G | 0.035 | ENST00000269305 | TP53 | missense_variant |
| **MM55** | chr11:108235900 | A | G | 0.337 | ENST00000278616 | ATM | missense_variant |
| **MM55** | chr2:239237343 | C | T | 0.016 | ENST00000373327 | TRAF3IP1 | missense_variant |
| **MM55** | chr7:2951887 | C | G | 0.615 | ENST00000396946 | CARD11 | missense_variant |
| **MM55** | chr9:127167651 | G | T | 0.505 | ENST00000259457 | PSMB7 | missense_variant |
| **MM56** | chr11:193845 | G | T | 0.26 | ENST00000342878 | SCGB1C1 | missense_variant |
| **MM56** | chr7:140453136 | A | T | 0.149 | ENST00000288602 | BRAF | missense_variant |
| **MM56** | chr17:62144293 | C | T | 0.08 | ENST00000433197 | ERN1 | splice_acceptor_variant |
| **MM56** | chr5:137802992 | C | T | 0.029 | ENST00000239938 | EGR1 | missense_variant |
| **MM57** | chr16:50826564 | TA | T | 0.646 | ENST00000311559 | CYLD | frameshift_variant |
| **MM58** | chr1:154402968 | A | C | 0.642 | ENST00000368485 | IL6R | missense_variant |
| **MM58** | chr2:231102955 | G | T | 0.21 | ENST00000392045 | SP140 | missense_variant |
| **MM59** | chr4:1807900 | C | T | 0.124 | ENST00000440486 | FGFR3 | splice_region_variant\|synony mous_variant |
| **MM59** | chr12:25398282 | C | G | 0.079 | ENST00000311936 | KRAS | missense_variant |
| **MM59** | chr13:73335951 | A | T | 0.148 | ENST00000377767 | DIS3 | splice_region_variant\|missens e_variant |
| **MM59** | chr13:73347935 | C | G | 0.403 | ENST00000377767 | DIS3 | missense_variant |
| **MM59** | chr19:38872779 | G | A | 0.716 | ENST00000215071 | PSMD8 | missense_variant |
| **MM59** | chr22:29196350 | GC | G | 0.444 | ENST00000216037 | XBP1 | frameshift_variant |
| **MM59** | chr5:137801686 | G | T | 0.354 | ENST00000239938 | EGR1 | missense_variant |
| **MM60** | chr12:25398284 | C | T | 0.016 | ENST00000311936 | KRAS | missense_variant |
| **MM60** | chr1:115258745 | C | G | 0.101 | ENST00000369535 | NRAS | missense_variant |
| **MM60** | chr1:115258747 | C | G | 0.016 | ENST00000369535 | NRAS | missense_variant |
| **MM60** | chr12:25380276 | T | A | 0.276 | ENST00000311936 | KRAS | missense_variant |
| **MM61** |  |  |  |  |  |  |  |
| **MM62** | chr12:25378647 | T | G | 0.459 | ENST00000311936 | KRAS | missense_variant |
| **MM62** | chr14:105945477 | C | T | 0.621 | ENST00000483017 | CRIP2 | missense_variant |
| **MM62** | chr12:25398284 | C | T | 0.025 | ENST00000311936 | KRAS | missense_variant |
| **MM62** | chr7:140453136 | A | T | 0.047 | ENST00000288602 | BRAF | missense_variant |
| **MM62** | chr19:39398300 | G | A | 0.585 | ENST00000313582 | NFKBIB | splice_donor_variant |

| **MM62** | chr1:154408560 | C | T | 0.616 | ENST00000368485 | IL6R | missense_variant |
| --- | --- | --- | --- | --- | --- | --- | --- |
| **MM62** | chr2:96810790 | G | A | 0.632 | ENST00000288943 | DUSP2 | missense_variant |
| **MM62** | chr14:35782179 | G | A | 0.425 | ENST00000261479 | PSMA6 | missense_variant |
| **MM62** | chr17:7578259 | A | T | 0.03 | ENST00000269305 | TP53 | missense_variant |
| **MM62** | chr4:106157182 | A | T | 0.046 | ENST00000380013 | TET2 | missense_variant |
| **MM63** | chr3:142261533 | T | C | 0.314 | ENST00000350721 | ATR | missense_variant |
| **MM63** | chr7:55249007 | G | A | 0.617 | ENST00000275493 | EGFR | missense_variant |
| **MM63** | chr1:118165814 | T | TA | 0.012 | ENST00000369448 | FAM46C | frameshift_variant |
| **MM63** | chr12:25398284 | C | A | 0.361 | ENST00000311936 | KRAS | missense_variant |
| **MM64** | chr1:161736167 | G | T | 0.549 | ENST00000367942 | ATF6 | missense_variant |
| **MM64** | chr13:49050977 | A | G | 0.41 | ENST00000267163 | RB1 | splice_region_variant\|synony mous_variant |
| **MM64** | chr5:137801711 | C | A | 0.408 | ENST00000239938 | EGR1 | missense_variant |
| **MM64** | chr6:37139016 | T | C | 0.353 | ENST00000373509 | PIM1 | missense_variant |
| **MM64** | chr6:37139039 | C | T | 0.338 | ENST00000373509 | PIM1 | stop_gained |
| **MM65** | chr1:115252240 | C | T | 0.016 | ENST00000369535 | NRAS | missense_variant |
| **MM65** | chr13:73336064 | C | T | 0.068 | ENST00000377767 | DIS3 | missense_variant |
| **MM65** | chr8:128753146 | G | A | 0.489 | ENST00000377970 | MYC | missense_variant |
| **MM66** | chr14:23511715 | C | A | 0.516 | ENST00000408907 | PSMB11 | missense_variant |
| **MM66** | chr8:77768114 | A | C | 0.361 | ENST00000521891 | ZFHX4 | missense_variant |
| **MM67** | chr16:50813626 | C | T | 0.504 | ENST00000311559 | CYLD | missense_variant |
| **MM67** | chr1:115256530 | G | T | 0.038 | ENST00000369535 | NRAS | missense_variant |
| **MM67** | chr12:25380275 | T | G | 0.236 | ENST00000311936 | KRAS | missense_variant |
| **MM68** | chr14:23512329 | A | G | 0.69 | ENST00000408907 | PSMB11 | missense_variant |
| **MM68** | chr12:25398284 | C | T | 0.062 | ENST00000311936 | KRAS | missense_variant |
| **MM68** | chr12:25398285 | C | A | 0.021 | ENST00000311936 | KRAS | missense_variant |
| **MM68** | chr1:115256529 | T | C | 0.093 | ENST00000369535 | NRAS | missense_variant |
| **MM68** | chr1:115256530 | G | T | 0.014 | ENST00000369535 | NRAS | missense_variant |
| **MM68** | chr12:25380275 | T | A | 0.015 | ENST00000311936 | KRAS | missense_variant |
| **MM68** | chr12:25380276 | T | C | 0.014 | ENST00000311936 | KRAS | missense_variant |
| **MM69** | chr12:25398285 | C | A | 0.027 | ENST00000311936 | KRAS | missense_variant |
| **MM69** | chr1:51439691 | ACTTTGCTGG | T | 0.377 | ENST00000371761 | CDKN2C | inframe_deletion\|stop_gained |
| **MM69** | chr1:51439715 | G | A | 0.438 | ENST00000371761 | CDKN2C | missense_variant |
| **MM69** | chr12:25380275 | T | G | 0.305 | ENST00000311936 | KRAS | missense_variant |
| **MM70** | chr2:231941856 | A | G | 0.438 | ENST00000308696 | PSMD1 | missense_variant |

| **MM70** | chr9:120475659 | T | C | 0.077 | ENST00000355622 | TLR4 | missense_variant |
| --- | --- | --- | --- | --- | --- | --- | --- |
| **MM71** | chr7:140453136 | A | T | 0.139 | ENST00000288602 | BRAF | missense_variant |
| **MM71** | chr1:118166435 | C | CG | 0.215 | ENST00000369448 | FAM46C | frameshift_variant |
| **MM72** | chr14:35871861 | A | T | 0.75 | ENST00000216797 | NFKBIA | stop_gained |
| **MM72** | chr14:103371876 | T | G | 0.817 | ENST00000560371 | TRAF3 | missense_variant |
| **MM72** | chr6:37138908 | G | C | 0.624 | ENST00000373509 | PIM1 | missense_variant |
| **MM72** | chr7:106508407 | A | G | 0.636 | ENST00000496166 | PIK3CG | missense_variant |
| **MM73** | chr12:25398284 | C | T | 0.443 | ENST00000311936 | KRAS | missense_variant |
| **MM73** | chr6:32825885 | G | A | 0.022 | ENST00000374859 | PSMB9 | missense_variant |
| **MM74** | chr12:25398285 | C | T | 0.479 | ENST00000311936 | KRAS | missense_variant |
| **MM74** | chr7:140481411 | C | T | 0.348 | ENST00000288602 | BRAF | missense_variant |
| **MM75** | chr15:99251184 | C | T | 0.013 | ENST00000268035 | IGF1R | missense_variant |
| **MM75** | chr1:118165863 | A | CAAGCTCAAA | 0.43 | ENST00000369448 | FAM46C | frameshift_variant |
| **MM75** | chr1:115256530 | G | T | 0.492 | ENST00000369535 | NRAS | missense_variant |
| **MM75** | chr2:54114548 | A | G | 0.469 | ENST00000404125 | PSME4 | missense_variant |
| **MM76** | chr22:29446562 | A | TCGGTGAGT | 0.63 | ENST00000544604 | ZNRF3 | inframe_insertion |
| **MM76** | chr12:25398284 | C | T | 0.007432 | ENST00000311936 | KRAS | missense_variant |
| **MM76** | chr12:25380275 | T | G | 0.021 | ENST00000311936 | KRAS | missense_variant |
| **MM76** | chr12:25398285 | C | T | 0.013 | ENST00000311936 | KRAS | missense_variant |
| **MM76** | chr3:142281823 | T | C | 0.672 | ENST00000350721 | ATR | missense_variant |
| **MM76** | chr6:37141718 | C | T | 0.028 | ENST00000373509 | PIM1 | missense_variant |
| **MM77** | chr17:7577121 | G | A | 0.293 | ENST00000269305 | TP53 | missense_variant |
| **MM77** | chr1:115258747 | C | T | 0.01 | ENST00000369535 | NRAS | missense_variant |
| **MM77** | chr14:103363685 | G | T | 0.55 | ENST00000560371 | TRAF3 | stop_gained |
| **MM78** | chr1:115256528 | T | G | 0.064 | ENST00000369535 | NRAS | missense_variant |
| **MM79** | chr1:118166047 | TCCTGGATTCT | T | 0.055 | ENST00000369448 | FAM46C | frameshift_variant |
| **MM79** | chr22:50355441 | G | A | 0.5 | ENST00000360612 | PIM3 | missense_variant |
| **MM79** | chr4:1803434 | G | C | 0.303 | ENST00000440486 | FGFR3 | missense_variant |
| **MM79** | chr9:139794122 | TCGG | T | 0.131 | ENST00000247668 | TRAF2 | splice_donor_variant |
| **MM80** | chr9:120476598 | G | A | 0.429 | ENST00000355622 | TLR4 | missense_variant |
| **MM80** | chr11:108201023 | T | C | 0.328 | ENST00000278616 | ATM | missense_variant |
| **MM80** | chr1:115256529 | T | C | 0.505 | ENST00000369535 | NRAS | missense_variant |
| **MM81** | chr11:108218080 | C | A | 0.355 | ENST00000278616 | ATM | missense_variant |
| **MM81** | chr12:25380275 | T | G | 0.449 | ENST00000311936 | KRAS | missense_variant |

| **MM82** | chr3:141328309 | CAGAA |  | C | 0.098 | ENST00000452898 | RASA2 | frameshift_variant |
| --- | --- | --- | --- | --- | --- | --- | --- | --- |
| **MM82** | chr3:142232440 | C |  | A | 0.651 | ENST00000350721 | ATR | missense_variant |
| **MM82** | chr5:137801463 | A |  | G | 0.411 | ENST00000239938 | EGR1 | missense_variant |
| **MM82** | chr7:106522610 | T |  | C | 0.587 | ENST00000496166 | PIK3CG | missense_variant |
| **MM83** | chr1:115256529 | T |  | C | 0.012 | ENST00000369535 | NRAS | missense_variant |
| **MM83** | chr1:118165967 | CAAG |  | C | 0.022 | ENST00000369448 | FAM46C | inframe_deletion |
| **MM83** | chr1:118166218 | G |  | GA | 0.049 | ENST00000369448 | FAM46C | frameshift_variant |
| **MM83** | chr10:104161233 | C |  | T | 0.18 | ENST00000369966 | NFKB2 | stop_gained |
| **MM83** | chr12:25398281 | C |  | T | 0.209 | ENST00000311936 | KRAS | missense_variant |
| **MM84** | chr12:25398284 | C |  | T | 0.08 | ENST00000311936 | KRAS | missense_variant |
| **MM84** | chr9:5077524 | A |  | C | 0.366 | ENST00000381652 | JAK2 | missense_variant |
| **MM84** | chr1:152187195 | G |  | A | 0.254 | ENST00000368801 | HRNR | stop_gained |
| **MM84** | chr4:106197402 | A |  | G | 0.037 | ENST00000380013 | TET2 | missense_variant |
| **MM84** | chr8:77766518 | A |  | T | 0.018 | ENST00000521891 | ZFHX4 | missense_variant |
| **MM85** | chr1:118165776 | C |  | CAT | 0.017 | ENST00000369448 | FAM46C | frameshift_variant |
| **MM85** | chr1:118165871 | CAA |  | C | 0.138 | ENST00000369448 | FAM46C | frameshift_variant |
| **MM85** | chr1:118166368 | AAAACCACTTC |  | A | 0.119 | ENST00000369448 | FAM46C | frameshift_variant |
| **MM85** | chr1:118166382 | G |  | A | 0.126 | ENST00000369448 | FAM46C | missense_variant |
| **MM85** | chr11:14539514 | T |  | G | 0.365 | ENST00000418988 | PSMA1 | missense_variant |
| **MM85** | chr13:48923105 | ATAAAT |  | A | 0.802 | ENST00000267163 | RB1 | frameshift_variant |
| **MM85** | chr14:103336737 | G |  | T | 0.11 | ENST00000560371 | TRAF3 | stop_gained |
| **MM85** | chr14:103371652 | GC |  | G | 0.255 | ENST00000560371 | TRAF3 | frameshift_variant |
| **MM85** | chr14:103371915 | CA |  | C | 0.047 | ENST00000560371 | TRAF3 | frameshift_variant |
| **MM85** | chr6:398827 | G |  | A | 0.458 | ENST00000380956 | IRF4 | splice_acceptor_variant |
| **MM85** | chr9:5022013 | C |  | T | 0.465 | ENST00000381652 | JAK2 | missense_variant |
| **MM86** |  |  |  |  |  |  |  |  |
| **MM87** | chr12:25378647 | T |  | G | 0.363 | ENST00000311936 | KRAS | missense_variant |
| **MM87** | chr13:113887610 | G |  | A | 0.561 | ENST00000375440 | CUL4A | missense_variant |
| **MM87** | chr1:118165743 | G |  | A | 0.484 | ENST00000369448 | FAM46C | missense_variant |
| **MM87** | chr12:25380275 | T |  | G | 0.054 | ENST00000311936 | KRAS | missense_variant |
| **MM87** | chr13:73346338 | C |  | T | 0.386 | ENST00000377767 | DIS3 | missense_variant |
| **MM87** | chr16:67969587 | C |  | A | 0.433 | ENST00000358514 | PSMB10 | missense_variant |
| **MM87** | chr6:47254156 | C |  | T | 0.52 | ENST00000296861 | TNFRSF21 | missense_variant |
| **MM87** | chr8:77761802 | A |  | G | 0.412 | ENST00000521891 | ZFHX4 | missense_variant |

| **MM88** | chr6:47200581 | C | T | 0.654 | ENST00000296861 | TNFRSF21 | missense_variant |
| --- | --- | --- | --- | --- | --- | --- | --- |
| **MM88** | chr1:115256528 | T | G | 0.257 | ENST00000369535 | NRAS | missense_variant |
| **MM89** | chr13:73346338 | C | T | 0.02 | ENST00000377767 | DIS3 | missense_variant |
| **MM89** | chr4:106196458 | C | G | 0.382 | ENST00000380013 | TET2 | missense_variant |
| **MM90** | chr1:115256530 | G | T | 0.368 | ENST00000369535 | NRAS | missense_variant |
| **MM90** | chr3:184025151 | T | C | 0.382 | ENST00000310118 | PSMD2 | missense_variant |
| **MM90** | chr6:106553126 | G | A | 0.587 | ENST00000369096 | PRDM1 | missense_variant |
| **MM91** | chr11:69456116 | C | G | 0.424 | ENST00000227507 | CCND1 | missense_variant |
| **MM91** | chr11:69456148 | C | G | 0.382 | ENST00000227507 | CCND1 | missense_variant |
| **MM91** | chr11:69456181 | G | T | 0.363 | ENST00000227507 | CCND1 | missense_variant |
| **MM91** | chr11:69456205 | G | A | 0.383 | ENST00000227507 | CCND1 | missense_variant |
| **MM91** | chr12:25398285 | C | G | 0.38 | ENST00000311936 | KRAS | missense_variant |
| **MM91** | chr4:1980558 | G | C | 0.51 | ENST00000382891 | WHSC1 | missense_variant |
| **MM92** | chr4:1980530 | C | T | 0.249 | ENST00000382891 | WHSC1 | missense_variant |
| **MM92** | chr7:140453136 | A | T | 0.296 | ENST00000288602 | BRAF | missense_variant |
| **MM92** | chr2:239237353 | G | C | 0.495 | ENST00000373327 | TRAF3IP1 | missense_variant |
| **MM92** | chr1:118166385 | GAA | G | 0.014 | ENST00000369448 | FAM46C | frameshift_variant |
| **MM93** | chr7:140453136 | A | T | 0.063 | ENST00000288602 | BRAF | missense_variant |
| **MM93** | chr12:25380275 | T | G | 0.118 | ENST00000311936 | KRAS | missense_variant |
| **MM93** | chr12:25398281 | C | T | 0.044 | ENST00000311936 | KRAS | missense_variant |
| **MM94** | chr1:115258745 | C | G | 0.389 | ENST00000369535 | NRAS | missense_variant |
| **MM94** | chr12:25380275 | T | G | 0.024 | ENST00000311936 | KRAS | missense_variant |
| **MM94** | chr6:394972 | A | G | 0.489 | ENST00000380956 | IRF4 | missense_variant |
| **MM94** | chr9:123605132 | A | T | 0.4 | ENST00000210313 | PSMD5 | missense_variant |
| **MM95** | chr1:115258747 | C | G | 0.02 | ENST00000369535 | NRAS | missense_variant |
| **MM95** | chr12:25398285 | C | T | 0.329 | ENST00000311936 | KRAS | missense_variant |
| **MM96** | chr3:184024201 | A | G | 0.305 | ENST00000310118 | PSMD2 | missense_variant |
| **MM96** | chr19:38869984 | G | A | 0.654 | ENST00000215071 | PSMD8 | missense_variant |
| **MM96** | chr12:25380275 | T | A | 0.283 | ENST00000311936 | KRAS | missense_variant |
| **MM96** | chr12:25398285 | C | T | 0.227 | ENST00000311936 | KRAS | missense_variant |
| **MM96** | chr12:92539148 | TGCTCACCTG | C | 0.5 | ENST00000256015 | BTG1 | splice_donor_variant |
| **MM96** | chr7:140453149 | C | G | 0.102 | ENST00000288602 | BRAF | missense_variant |
| **MM96** | chr7:140453155 | C | T | 0.051 | ENST00000288602 | BRAF | missense_variant |
| **MM97** | chr11:14539222 | T | C | 0.501 | ENST00000418988 | PSMA1 | missense_variant |

| **MM97** | chr19:18279643 | A | G | 0.497 | ENST00000222254 | PIK3R2 | missense_variant |
| --- | --- | --- | --- | --- | --- | --- | --- |
| **MM97** | chr5:137801691 | A | G | 0.731 | ENST00000239938 | EGR1 | missense_variant |
| **MM98** | chr12:25378647 | T | G | 0.021 | ENST00000311936 | KRAS | missense_variant |
| **MM98** | chr12:25398284 | C | T | 0.422 | ENST00000311936 | KRAS | missense_variant |
| **MM98** | chr14:65560490 | C | T | 0.167 | ENST00000358664 | MAX | missense_variant |
| **MM98** | chr14:65560491 | T | A | 0.092 | ENST00000358664 | MAX | missense_variant |
| **MM98** | chr14:65560524 | G | A | 0.111 | ENST00000358664 | MAX | missense_variant |
| **MM98** | chr17:79479083 | G | C | 0.446 | ENST00000331925 | ACTG1 | missense_variant |
| **MM98** | chr2:88926581 | G | C | 0.464 | ENST00000303236 | EIF2AK3 | missense_variant |
| **MM99** | chr1:115256529 | T | C | 0.02 | ENST00000369535 | NRAS | missense_variant |
| **MM99** | chr1:115256530 | G | T | 0.216 | ENST00000369535 | NRAS | missense_variant |
| **MM100** | chr1:115258748 | C | A | 0.019 | ENST00000369535 | NRAS | missense_variant |
| **MM100** | chr10:104156660 | G | C | 0.436 | ENST00000369966 | NFKB2 | splice_acceptor_variant |
| **MM100** | chr10:104160414 | CTG | C | 0.585 | ENST00000369966 | NFKB2 | frameshift_variant |
| **MM100** | chr12:25398285 | C | G | 0.775 | ENST00000311936 | KRAS | missense_variant |
| **MM101** | chr7:140453136 | A | T | 0.383 | ENST00000288602 | BRAF | missense_variant |
| **MM101** | chr11:69456101 | G | A | 0.31 | ENST00000227507 | CCND1 | missense_variant |
| **MM101** | chr16:50783996 | TC | T | 0.48 | ENST00000311559 | CYLD | frameshift_variant |
| **MM101** | chr3:142274752 | G | T | 0.04 | ENST00000350721 | ATR | missense_variant |
| **MM102** |  |  |  |  |  |  |  |
| **MM103** | chr6:32809404 | G | A | 0.255 | ENST00000374881 | PSMB8 | missense_variant |
| **MM103** | chr1:118166319 | G | A | 0.028 | ENST00000369448 | FAM46C | missense_variant |
| **MM103** | chr13:73336065 | T | C | 0.026 | ENST00000377767 | DIS3 | missense_variant |
| **MM103** | chr13:73346338 | C | T | 0.243 | ENST00000377767 | DIS3 | missense_variant |
| **MM103** | chr14:35872389 | G | A | 0.19 | ENST00000216797 | NFKBIA | missense_variant |
| **MM103** | chr14:53184823 | G | A | 0.024 | ENST00000606149 | PSMC6 | missense_variant |
| **MM103** | chr17:7577085 | C | T | 0.069 | ENST00000269305 | TP53 | missense_variant |
| **MM103** | chr17:7577099 | C | G | 0.034 | ENST00000269305 | TP53 | missense_variant |
| **MM103** | chr17:7577509 | C | G | 0.041 | ENST00000269305 | TP53 | missense_variant |
| **MM103** | chr2:162247624 | C | T | 0.023 | ENST00000409682 | PSMD14 | missense_variant |
| **MM103** | chr2:231174720 | G | T | 0.148 | ENST00000392045 | SP140 | stop_gained |
| **MM103** | chr4:106197197 | G | A | 0.189 | ENST00000380013 | TET2 | missense_variant |
| **MM103** | chr7:140481402 | C | A | 0.016 | ENST00000288602 | BRAF | missense_variant |
| **MM104** | chr12:25380275 | T | A | 0.011 | ENST00000311936 | KRAS | missense_variant |

| **MM104** | chr12:25380276 | T |  | C | 0.297 | ENST00000311936 | KRAS | missense_variant |
| --- | --- | --- | --- | --- | --- | --- | --- | --- |
| **MM105** | chr2:231134245 | A |  | C | 0.153 | ENST00000392045 | SP140 | splice_acceptor_variant |
| **MM106** | chr16:67970596 | C |  | T | 0.442 | ENST00000358514 | PSMB10 | splice_donor_variant |
| **MM106** | chr9:21974794 | ATGCTGCTCCC |  | A | 0.267 | ENST00000304494 | CDKN2A | inframe_deletion |
| **MM106** | chr1:118165864 | A |  | AC | 0.183 | ENST00000369448 | FAM46C | frameshift_variant |
| **MM107** | chr11:108224608 | G |  | A | 0.346 | ENST00000278616 | ATM | splice_donor_variant |
| **MM107** | chr1:118165901 | A |  | AT | 0.077 | ENST00000369448 | FAM46C | frameshift_variant |
| **MM107** | chr12:25398281 | C |  | T | 0.48 | ENST00000311936 | KRAS | missense_variant |
| **MM107** | chr3:142281578 | A |  | T | 0.409 | ENST00000350721 | ATR | missense_variant |
| **MM108** | chr1:115252240 | C |  | T | 0.016 | ENST00000369535 | NRAS | missense_variant |
| **MM108** | chr14:35871746 | AG |  | A | 0.114 | ENST00000216797 | NFKBIA | frameshift_variant |
| **MM108** | chr6:106553560 | G |  | T | 0.243 | ENST00000369096 | PRDM1 | missense_variant |
| **MM109** | chr5:137803274 | G |  | A | 0.033 | ENST00000239938 | EGR1 | missense_variant |
| **MM110** | chr7:140453136 | A |  | T | 0.458 | ENST00000288602 | BRAF | missense_variant |
| **MM110** | chr15:99456337 | G |  | A | 0.498 | ENST00000268035 | IGF1R | missense_variant |
| **MM111** | chr1:115258745 | C |  | G | 0.215 | ENST00000369535 | NRAS | missense_variant |
| **MM111** | chr1:115258748 | C |  | T | 0.016 | ENST00000369535 | NRAS | missense_variant |
| **MM112** | chr1:115258745 | C |  | A | 0.06 | ENST00000369535 | NRAS | missense_variant |
| **MM112** | chr1:118166302 | GCTC |  | G | 0.488 | ENST00000369448 | FAM46C | inframe_deletion |
| **MM112** | chr1:118166500 | C |  | T | 0.481 | ENST00000369448 | FAM46C | missense_variant |
| **MM112** | chr12:112888199 | C |  | T | 0.065 | ENST00000351677 | PTPN11 | missense_variant |
| **MM112** | chr16:50815206 | A |  | G | 0.02 | ENST00000311559 | CYLD | missense_variant |
| **MM112** | chr2:136872485 | G |  | C | 0.053 | ENST00000241393 | CXCR4 | stop_gained |
| **MM113** | chr1:115251203 | C |  | A | 0.015 | ENST00000369535 | NRAS | missense_variant |
| **MM113** | chr1:115258745 | C |  | A | 0.171 | ENST00000369535 | NRAS | missense_variant |
| **MM113** | chr12:25398282 | C |  | G | 0.123 | ENST00000311936 | KRAS | missense_variant |
| **MM113** | chr12:25398284 | C |  | G | 0.024 | ENST00000311936 | KRAS | missense_variant |
| **MM113** | chr4:106157573 | C |  | T | 0.036 | ENST00000380013 | TET2 | missense_variant |
| **MM113** | chr6:106555065 | G |  | T | 0.509 | ENST00000369096 | PRDM1 | stop_gained |
| **MM114** | chr12:25378647 | T |  | G | 0.00813 | ENST00000311936 | KRAS | missense_variant |
| **MM114** | chr17:7574018 | G |  | A | 0.023 | ENST00000269305 | TP53 | missense_variant |
| **MM114** | chr11:102195997 | C |  | T | 0.012 | ENST00000263464 | BIRC3 | stop_gained |
| **MM114** | chr12:25362838 | T |  | A | 0.061 | ENST00000311936 | KRAS | missense_variant |
| **MM114** | chr12:25362841 | A |  | C | 0.066 | ENST00000311936 | KRAS | missense_variant |

| **MM114** | chr12:25380276 | T | A | 0.013 | ENST00000311936 | KRAS | missense_variant |
| --- | --- | --- | --- | --- | --- | --- | --- |
| **MM114** | chr12:25380282 | G | C | 0.02 | ENST00000311936 | KRAS | missense_variant |
| **MM114** | chr12:25398281 | C | T | 0.009901 | ENST00000311936 | KRAS | missense_variant |
| **MM114** | chr17:7578203 | C | T | 0.033 | ENST00000269305 | TP53 | missense_variant |
| **MM114** | chr3:30691871 | G | A | 0.013 | ENST00000295754 | TGFBR2 | missense_variant |
| **MM114** | chr7:140453154 | T | C | 0.012 | ENST00000288602 | BRAF | missense_variant |
| **MM115** | chr1:115256528 | T | G | 0.142 | ENST00000369535 | NRAS | missense_variant |
| **MM115** | chr1:118166213 | C | CG | 0.018 | ENST00000369448 | FAM46C | frameshift_variant |
| **MM115** | chr3:141327349 | AC | A | 0.624 | ENST00000452898 | RASA2 | frameshift_variant |
| **MM115** | chr7:50367287 | C | T | 0.017 | ENST00000331340 | IKZF1 | missense_variant |
| **MM116** | chr1:115256529 | T | C | 0.322 | ENST00000369535 | NRAS | missense_variant |
| **MM117** | chr1:115258747 | C | T | 0.434 | ENST00000369535 | NRAS | missense_variant |
| **MM117** | chr1:115252240 | C | T | 0.014 | ENST00000369535 | NRAS | missense_variant |
| **MM117** | chr1:118165919 | G | GA | 0.068 | ENST00000369448 | FAM46C | frameshift_variant |
| **MM118** | chr7:106508067 | C | T | 0.543 | ENST00000496166 | PIK3CG | missense_variant |
| **MM118** | chr17:16842964 | C | T | 0.581 | ENST00000261652 | TNFRSF13B | missense_variant |
| **MM118** | chr11:108201023 | T | C | 0.509 | ENST00000278616 | ATM | missense_variant |
| **MM119** | chr22:29196469 | G | A | 0.55 | ENST00000216037 | XBP1 | missense_variant |
| **MM119** | chr1:118166339 | A | AC | 0.864 | ENST00000369448 | FAM46C | frameshift_variant |
| **MM119** | chr2:231113641 | G | T | 0.014 | ENST00000392045 | SP140 | stop_gained |
| **MM120** | chr15:99500531 | G | A | 0.566 | ENST00000268035 | IGF1R | missense_variant |
| **MM120** | chr16:50813914 | C | T | 0.048 | ENST00000311559 | CYLD | stop_gained |
| **MM120** | chr16:50828194 | G | A | 0.029 | ENST00000311559 | CYLD | stop_gained |
| **MM120** | chr22:23230313 | T | C | 0.825 | ENST00000526893 | IGLL5 | missense_variant |
| **MM120** | chr9:120475233 | C | T | 0.012 | ENST00000355622 | TLR4 | missense_variant |
| **MM121** | chr1:154408560 | C | T | 0.486 | ENST00000368485 | IL6R | missense_variant |
| **MM121** | chr1:115256530 | G | T | 0.415 | ENST00000369535 | NRAS | missense_variant |
| **MM121** | chr13:73336068 | T | A | 0.371 | ENST00000377767 | DIS3 | missense_variant |
| **MM121** | chr13:73346338 | C | T | 0.015 | ENST00000377767 | DIS3 | missense_variant |
| **MM121** | chr14:65560458 | G | A | 0.022 | ENST00000358664 | MAX | missense_variant |
| **MM121** | chr5:137801574 | AGCAACG | A | 0.558 | ENST00000239938 | EGR1 | inframe_deletion |
| **MM122** | chr21:43161696 | C | T | 0.203 | ENST00000332512 | RIPK4 | missense_variant |
| **MM122** | chr13:73336136 | G | A | 0.397 | ENST00000377767 | DIS3 | missense_variant |
| **MM123** | chr7:140453136 | A | T | 0.308 | ENST00000288602 | BRAF | missense_variant |

| **MM123** | chr1:118165774 | TC | T | 0.037 | ENST00000369448 | FAM46C | frameshift_variant |
| --- | --- | --- | --- | --- | --- | --- | --- |
| **MM123** | chr13:73346338 | C | T | 0.4 | ENST00000377767 | DIS3 | missense_variant |
| **MM124** | chr2:96810790 | G | A | 0.583 | ENST00000288943 | DUSP2 | missense_variant |
| **MM124** | chr14:58727716 | T | C | 0.261 | ENST00000216455 | PSMA3 | missense_variant |
| **MM124** | chr5:142780213 | A | C | 0.509 | ENST00000343796 | NR3C1 | missense_variant |
| **MM125** | chr1:115258747 | C | A | 0.171 | ENST00000369535 | NRAS | missense_variant |
| **MM125** | chr1:144865846 | G | A | 0.318 | ENST00000369356 | PDE4DIP | stop_gained |
| **MM125** | chr13:73355010 | G | C | 0.51 | ENST00000377767 | DIS3 | missense_variant |
| **MM125** | chr3:64008978 | C | T | 0.565 | ENST00000492933 | PSMD6 | missense_variant |
| **MM125** | chr7:140453155 | C | T | 0.332 | ENST00000288602 | BRAF | missense_variant |
| **MM126** | chr14:103371968 | CA | C | 0.508 | ENST00000560371 | TRAF3 | frameshift_variant |
| **MM127** | chr2:96810582 | C | T | 0.441 | ENST00000288943 | DUSP2 | missense_variant |
| **MM128** | chr12:25398284 | C | T | 0.41 | ENST00000311936 | KRAS | missense_variant |
| **MM128** | chr1:118165603 | G | A | 0.487 | ENST00000369448 | FAM46C | missense_variant |
| **MM128** | chr3:30691871 | G | A | 0.015 | ENST00000295754 | TGFBR2 | missense_variant |
| **MM128** | chr8:77764565 | A | C | 0.475 | ENST00000521891 | ZFHX4 | missense_variant |
| **MM129** | chr11:69456214 | T | G | 0.595 | ENST00000227507 | CCND1 | missense_variant |
| **MM129** | chr3:141289866 | T | A | 0.016 | ENST00000452898 | RASA2 | missense_variant |
| **MM130** | chr17:7579358 | C | T | 0.467 | ENST00000269305 | TP53 | missense_variant |
| **MM130** | chr8:128750527 | T | C | 0.452 | ENST00000377970 | MYC | missense_variant |
| **MM130** | chr12:49087460 | G | A | 0.518 | ENST00000261900 | CCNT1 | missense_variant |
| **MM130** | chr7:106508418 | G | A | 0.372 | ENST00000496166 | PIK3CG | missense_variant |
| **MM130** | chr12:112926888 | G | T | 0.026 | ENST00000351677 | PTPN11 | missense_variant |
| **MM130** | chr7:140453155 | C | T | 0.011 | ENST00000288602 | BRAF | missense_variant |
| **MM131** | chr14:103371824 | C | A | 0.081 | ENST00000560371 | TRAF3 | missense_variant |
| **MM131** | chr9:139815668 | G | A | 0.071 | ENST00000247668 | TRAF2 | splice_donor_variant |
| **MM132** | chr1:115258744 | C | T | 0.983 | ENST00000369535 | NRAS | missense_variant |
| **MM132** | chr1:118166039 | TTTC | T | 0.955 | ENST00000369448 | FAM46C | inframe_deletion |
| **MM132** | chr1:118166043 | C | A | 0.959 | ENST00000369448 | FAM46C | missense_variant |
| **MM132** | chr11:108172500 | G | A | 0.456 | ENST00000278616 | ATM | missense_variant |
| **MM132** | chr14:105995384 | G | C | 0.466 | ENST00000392519 | TMEM121 | missense_variant |
| **MM132** | chr17:37933905 | A | G | 0.475 | ENST00000346872 | IKZF3 | splice_region_variant\|synony mous_variant |
| **MM132** | chr17:37933948 | C | G | 0.492 | ENST00000346872 | IKZF3 | missense_variant |
| **MM132** | chr17:37933966 | A | C | 0.496 | ENST00000346872 | IKZF3 | missense_variant |

| **MM132** | chr3:142281172 | G | A | 0.014 | ENST00000350721 | ATR | missense_variant |
| --- | --- | --- | --- | --- | --- | --- | --- |
| **MM133** | chr12:25398284 | C | A | 0.314 | ENST00000311936 | KRAS | missense_variant |
| **MM134** | chr12:25380275 | T | G | 0.016 | ENST00000311936 | KRAS | missense_variant |
| **MM134** | chr7:50468174 | T | G | 0.026 | ENST00000331340 | IKZF1 | missense_variant |
| **MM135** | chr9:139793371 | G | A | 0.481 | ENST00000247668 | TRAF2 | missense_variant |
| **MM136** | chr11:108114835 | C | T | 0.457 | ENST00000278616 | ATM | stop_gained |
| **MM136** | chr11:108143539 | C | CTG | 0.544 | ENST00000278616 | ATM | frameshift_variant |
| **MM136** | chr11:108143541 | TC | T | 0.54 | ENST00000278616 | ATM | frameshift_variant |
| **MM136** | chr4:106196948 | C | T | 0.531 | ENST00000380013 | TET2 | missense_variant |
| **MM136** | chr7:55220278 | G | T | 0.343 | ENST00000275493 | EGFR | missense_variant |
| **MM137** | chr1:118166309 | GTTC | G | 0.056 | ENST00000369448 | FAM46C | inframe_deletion |
| **MM138** | chr14:35872045 | CTAAGTG | C | 0.304 | ENST00000216797 | NFKBIA | inframe_deletion |
| **MM138** | chr14:35872054 | G | C | 0.319 | ENST00000216797 | NFKBIA | missense_variant |
| **MM138** | chr14:105996097 | T | C | 0.532 | ENST00000392519 | TMEM121 | missense_variant |
| **MM138** | chr2:209113113 | G | A | 0.383 | ENST00000446179 | IDH1 | missense_variant |
| **MM139** | chr12:25380276 | T | A | 0.456 | ENST00000311936 | KRAS | missense_variant |
| **MM139** | chr6:394972 | A | G | 0.394 | ENST00000380956 | IRF4 | missense_variant |
| **MM140** | chr12:25380275 | T | G | 0.28 | ENST00000311936 | KRAS | missense_variant |
| **MM140** | chr13:73355010 | G | C | 0.889 | ENST00000377767 | DIS3 | missense_variant |
| **MM141** | chr2:96810609 | C | T | 0.476 | ENST00000288943 | DUSP2 | missense_variant |
| **MM141** | chr3:142266590 | C | T | 0.456 | ENST00000350721 | ATR | missense_variant |
| **MM141** | chr11:108121752 | CAG | C | 0.629 | ENST00000278616 | ATM | frameshift_variant |
| **MM141** | chr1:115256529 | T | A | 0.596 | ENST00000369535 | NRAS | missense_variant |
| **MM141** | chr2:96810802 | C | T | 0.643 | ENST00000288943 | DUSP2 | missense_variant |
| **MM141** | chr5:137801743 | A | G | 0.6 | ENST00000239938 | EGR1 | missense_variant |
| **MM142** | chr4:15835912 | C | T | 0.993 | ENST00000226279 | CD38 | missense_variant |
| **MM142** | chr5:142680227 | T | C | 0.665 | ENST00000343796 | NR3C1 | missense_variant |
| **MM142** | chr5:55264223 | C | T | 0.653 | ENST00000381298 | IL6ST | splice_region_variant\|synony mous_variant |
| **MM142** | chr17:16852292 | G | GT | 0.469 | ENST00000261652 | TNFRSF13B | frameshift_variant |
| **MM142** | chr1:115256528 | T | A | 0.38 | ENST00000369535 | NRAS | missense_variant |
| **MM142** | chr2:96810528 | G | C | 0.563 | ENST00000288943 | DUSP2 | missense_variant |
| **MM143** | chr1:115256528 | T | A | 0.061 | ENST00000369535 | NRAS | missense_variant |
| **MM143** | chr12:12871036 | A | G | 0.167 | ENST00000228872 | CDKN1B | missense_variant |
| **MM143** | chr12:25398284 | C | A | 0.019 | ENST00000311936 | KRAS | missense_variant |

| **MM144** | chr1:118166420 | TC | T | 0.166 | ENST00000369448 | FAM46C | frameshift_variant |
| --- | --- | --- | --- | --- | --- | --- | --- |
| **MM144** | chr12:25380275 | T | A | 0.216 | ENST00000311936 | KRAS | missense_variant |
| **MM145** | chr1:115258744 | C | T | 0.444 | ENST00000369535 | NRAS | missense_variant |
| **MM145** | chr13:73337723 | C | T | 0.533 | ENST00000377767 | DIS3 | missense_variant |
| **MM145** | chr14:103357674 | C | T | 0.539 | ENST00000560371 | TRAF3 | stop_gained |
| **MM145** | chr17:7576855 | G | A | 0.306 | ENST00000269305 | TP53 | splice_region_variant\|stop_ga ined |
| **MM146** | chr17:40497584 | G | A | 0.472 | ENST00000264657 | STAT3 | missense_variant |
| **MM146** | chr19:18266991 | G | A | 0.5 | ENST00000222254 | PIK3R2 | missense_variant |
| **MM146** | chr14:35871779 | CA | C | 0.166 | ENST00000216797 | NFKBIA | frameshift_variant |
| **MM146** | chr14:35872499 | C | T | 0.18 | ENST00000216797 | NFKBIA | missense_variant |
| **MM147** | chr17:61909292 | G | A | 0.448 | ENST00000310144 | PSMC5 | missense_variant |
| **MM147** | chr17:7577141 | C | A | 0.142 | ENST00000269305 | TP53 | missense_variant |
| **MM147** | chr17:7578271 | T | A | 0.41 | ENST00000269305 | TP53 | missense_variant |
| **MM147** | chr7:140453134 | T | C | 0.016 | ENST00000288602 | BRAF | missense_variant |
| **MM148** | chr17:79479166 | G | A | 0.432 | ENST00000331925 | ACTG1 | splice_region_variant\|synony mous_variant |
| **MM148** | chr3:141295907 | G | T | 0.482 | ENST00000452898 | RASA2 | missense_variant |
| **MM148** | chr13:73336064 | C | T | 0.192 | ENST00000377767 | DIS3 | missense_variant |
| **MM148** | chr7:50467783 | G | T | 0.39 | ENST00000331340 | IKZF1 | missense_variant |
| **MM149** | chr9:120470895 | G | A | 0.066 | ENST00000355622 | TLR4 | missense_variant |
| **MM149** | chr12:25398285 | C | T | 0.071 | ENST00000311936 | KRAS | missense_variant |
| **MM150** | chr21:43161407 | T | A | 0.352 | ENST00000332512 | RIPK4 | missense_variant |
| **MM151** | chr1:115256530 | G | T | 0.103 | ENST00000369535 | NRAS | missense_variant |
| **MM152** | chr3:42787467 | G | A | 0.587 | ENST00000310232 | CCDC13 | missense_variant |
| **MM152** | chr3:142257354 | G | T | 0.519 | ENST00000350721 | ATR | missense_variant |
| **MM152** | chr2:54135661 | A | G | 0.435 | ENST00000404125 | PSME4 | missense_variant |
| **MM153** | chr20:39317434 | C | G | 0.316 | ENST00000373313 | MAFB | missense_variant |
| **MM154** | chr12:25398281 | C | T | 0.462 | ENST00000311936 | KRAS | missense_variant |
| **MM155** | chr1:115258747 | C | G | 0.409 | ENST00000369535 | NRAS | missense_variant |
| **MM155** | chr2:88882948 | C | T | 0.494 | ENST00000303236 | EIF2AK3 | splice_region_variant\|missens e_variant |
| **MM156** | chr1:151373777 | A | C | 0.513 | ENST00000290541 | PSMB4 | missense_variant |
| **MM156** | chr12:112888210 | G | A | 0.45 | ENST00000351677 | PTPN11 | missense_variant |
| **MM157** | chr12:25378647 | T | G | 0.251 | ENST00000311936 | KRAS | missense_variant |
| **MM157** | chr11:108173592 | C | T | 0.543 | ENST00000278616 | ATM | missense_variant |

| **MM157** | chr22:23230432 | T |  | C | 0.262 | ENST00000526893 | IGLL5 | missense_variant |
| --- | --- | --- | --- | --- | --- | --- | --- | --- |
| **MM158** | chr11:14632507 | C |  | T | 0.532 | ENST00000418988 | PSMA1 | splice_donor_5th_base_varian t |
| **MM158** | chr12:25398284 | C |  | T | 0.036 | ENST00000311936 | KRAS | missense_variant |
| **MM158** | chr12:25380275 | T |  | G | 0.318 | ENST00000311936 | KRAS | missense_variant |
| **MM159** | chr1:115256529 | T |  | C | 0.084 | ENST00000369535 | NRAS | missense_variant |
| **MM159** | chr14:103336615 | TGCTGGGACG |  | G | 0.397 | ENST00000560371 | TRAF3 | frameshift_variant |
| **MM159** | chr16:50785778 | AG |  | A | 0.19 | ENST00000311559 | CYLD | frameshift_variant |
| **MM159** | chr2:231152633 | ACTCAGAGTG |  | C | 0.11 | ENST00000392045 | SP140 | frameshift_variant |
| **MM160** | chr11:108122617 | C |  | T | 0.33 | ENST00000278616 | ATM | missense_variant |
| **MM160** | chr17:7579410 | G |  | T | 0.268 | ENST00000269305 | TP53 | missense_variant |
| **MM160** | chr5:55237118 | T |  | C | 0.545 | ENST00000381298 | IL6ST | missense_variant |
| **MM161** | chr14:105995576 | G |  | C | 0.308 | ENST00000392519 | TMEM121 | missense_variant |
| **MM161** | chr22:23230278 | G |  | C | 0.221 | ENST00000526893 | IGLL5 | missense_variant |
| **MM161** | chr7:55268044 | C |  | T | 0.345 | ENST00000275493 | EGFR | missense_variant |
| **MM161** | chr1:118166359 | C |  | CT | 0.262 | ENST00000369448 | FAM46C | frameshift_variant |
| **MM161** | chr1:118166362 | A |  | G | 0.254 | ENST00000369448 | FAM46C | missense_variant |
| **MM161** | chr1:118166449 | G |  | C | 0.305 | ENST00000369448 | FAM46C | missense_variant |
| **MM162** | chr12:49087376 | G |  | A | 0.474 | ENST00000261900 | CCNT1 | missense_variant |
| **MM162** | chr10:104157337 | G |  | C | 0.034 | ENST00000369966 | NFKB2 | missense_variant |
| **MM163** | chr8:77766175 | G |  | A | 0.401 | ENST00000521891 | ZFHX4 | missense_variant |
| **MM164** | chr1:53158533 | T |  | C | 0.414 | ENST00000371538 | COA7 | missense_variant |
| **MM164** | chr17:7578221 | TTC |  | T | 0.387 | ENST00000269305 | TP53 | frameshift_variant |
| **MM164** | chr17:40475030 | T |  | G | 0.491 | ENST00000264657 | STAT3 | missense_variant |
| **MM165** | chr1:115256529 | T |  | A | 0.43 | ENST00000369535 | NRAS | missense_variant |
| **MM165** | chr11:108216528 | A |  | C | 0.054 | ENST00000278616 | ATM | missense_variant |
| **MM165** | chr3:64008346 | T |  | C | 0.48 | ENST00000492933 | PSMD6 | missense_variant |
| **MM166** | chr2:54161862 | T |  | A | 0.461 | ENST00000404125 | PSME4 | splice_region_variant\|synony mous_variant |
| **MM166** | chr8:77763229 | T |  | A | 0.419 | ENST00000521891 | ZFHX4 | missense_variant |
| **MM166** | chrX:119675569 | T |  | C | 0.283 | ENST00000404115 | CUL4B | missense_variant |
| **MM167** | chr13:73346340 | T |  | A | 0.061 | ENST00000377767 | DIS3 | missense_variant |
| **MM167** | chr14:105995472 | C |  | G | 0.681 | ENST00000392519 | TMEM121 | missense_variant |
| **MM168** | chr1:115256528 | T |  | G | 0.024 | ENST00000369535 | NRAS | missense_variant |
| **MM168** | chr11:108143450 | C |  | A | 0.021 | ENST00000278616 | ATM | splice_region_variant\|missens e_variant |

| **MM169** | chr1:118165567 | A | C | 0.399 | ENST00000369448 | FAM46C | missense_variant |
| --- | --- | --- | --- | --- | --- | --- | --- |
| **MM169** | chr16:50825471 | C | G | 0.081 | ENST00000311559 | CYLD | splice_region_variant\|stop_ga ined |
| **MM169** | chr16:50826545 | G | A | 0.257 | ENST00000311559 | CYLD | missense_variant |
| **MM169** | chr6:394920 | G | T | 0.417 | ENST00000380956 | IRF4 | missense_variant |
| **MM169** | chr9:128003284 | C | T | 0.181 | ENST00000324460 | HSPA5 | splice_region_variant\|missens e_variant |
| **MM170** | chr12:25398285 | C | T | 0.045 | ENST00000311936 | KRAS | missense_variant |
| **MM170** | chr13:49039464 | G | T | 0.014 | ENST00000267163 | RB1 | stop_gained |
| **MM170** | chr14:103371823 | A | G | 0.019 | ENST00000560371 | TRAF3 | missense_variant |
| **MM171** | chr17:62122734 | C | T | 0.952 | ENST00000433197 | ERN1 | missense_variant |
| **MM171** | chr14:103338252 | A | C | 0.034 | ENST00000560371 | TRAF3 | splice_acceptor_variant |
| **MM171** | chr14:103371880 | C | T | 0.1 | ENST00000560371 | TRAF3 | missense_variant |
| **MM171** | chr14:103372042 | T | C | 0.088 | ENST00000560371 | TRAF3 | missense_variant |
| **MM171** | chr17:7574003 | G | A | 0.158 | ENST00000269305 | TP53 | stop_gained |
| **MM171** | chr17:7577571 | ATGTAGT | A | 0.317 | ENST00000269305 | TP53 | inframe_deletion |
| **MM171** | chr17:7578221 | TTC | T | 0.031 | ENST00000269305 | TP53 | frameshift_variant |
| **MM171** | chr17:7578429 | CTG | C | 0.086 | ENST00000269305 | TP53 | frameshift_variant |
| **MM171** | chr4:15835907 | G | T | 0.129 | ENST00000226279 | CD38 | missense_variant |
| **MM172** | chr1:115256529 | T | C | 0.227 | ENST00000369535 | NRAS | missense_variant |
| **MM172** | chr17:7576868 | T | TTC | 0.247 | ENST00000269305 | TP53 | frameshift_variant |
| **MM172** | chr17:7579312 | C | A | 0.378 | ENST00000269305 | TP53 | splice_region_variant\|synony mous_variant |
| **MM173** | chr13:73346341 | C | G | 0.377 | ENST00000377767 | DIS3 | missense_variant |
| **MM173** | chr3:141295897 | TGCTGTAGCC | T | 0.352 | ENST00000452898 | RASA2 | inframe_deletion |
| **MM173** | chr7:140481411 | C | T | 0.057 | ENST00000288602 | BRAF | missense_variant |
| **MM174** | chr1:115256529 | T | A | 0.219 | ENST00000369535 | NRAS | missense_variant |
| **MM175** | chr12:25380276 | T | C | 0.371 | ENST00000311936 | KRAS | missense_variant |
| **MM175** | chr14:65560490 | C | T | 0.819 | ENST00000358664 | MAX | missense_variant |
| **MM175** | chr19:18271335 | C | T | 0.587 | ENST00000222254 | PIK3R2 | missense_variant |
| **MM176** | chr12:112926888 | G | T | 0.463 | ENST00000351677 | PTPN11 | missense_variant |
| **MM177** | chr17:7578406 | C | T | 0.986 | ENST00000269305 | TP53 | missense_variant |
| **MM177** | chr1:118165711 | C | T | 0.988 | ENST00000369448 | FAM46C | missense_variant |
| **MM177** | chr11:108180939 | C | G | 0.477 | ENST00000278616 | ATM | missense_variant |
| **MM178** | chr21:34799310 | C | T | 0.451 | ENST00000290219 | IFNGR2 | missense_variant |
| **MM178** | chr7:140453136 | A | T | 0.084 | ENST00000288602 | BRAF | missense_variant |

| **MM178** | chr14:35871860 | T | C | 0.144 | ENST00000216797 | NFKBIA | missense_variant |
| --- | --- | --- | --- | --- | --- | --- | --- |
| **MM178** | chr17:7578554 | A | T | 0.098 | ENST00000269305 | TP53 | splice_region_variant\|missens e_variant |
| **MM179** | chr1:115256529 | T | C | 0.182 | ENST00000369535 | NRAS | missense_variant |
| **MM179** | chr1:115258745 | C | G | 0.065 | ENST00000369535 | NRAS | missense_variant |
| **MM179** | chr1:118166114 | CACCGTGA | C | 0.17 | ENST00000369448 | FAM46C | frameshift_variant |
| **MM179** | chr12:25398281 | C | T | 0.051 | ENST00000311936 | KRAS | missense_variant |
| **MM179** | chr22:23230364 | C | G | 0.464 | ENST00000526893 | IGLL5 | missense_variant |
| **MM180** | chr13:73355087 | G | A | 0.22 | ENST00000377767 | DIS3 | missense_variant |
| **MM180** | chr14:105932775 | G | A | 0.489 | ENST00000331320 | MTA1 | missense_variant |
| **MM180** | chr22:29192045 | G | T | 0.178 | ENST00000216037 | XBP1 | missense_variant |
| **MM181** | chr22:29191649 | A | G | 0.152 | ENST00000216037 | XBP1 | missense_variant |
| **MM181** | chr12:25380275 | T | G | 0.377 | ENST00000311936 | KRAS | missense_variant |
| **MM181** | chr2:162242035 | A | G | 0.421 | ENST00000409682 | PSMD14 | missense_variant |
| **MM182** | chr14:90726534 | G | A | 0.678 | ENST00000261303 | PSMC1 | missense_variant |
| **MM182** | chr1:118165727 | TTTGGTCAA | T | 0.023 | ENST00000369448 | FAM46C | frameshift_variant |
| **MM182** | chr22:29195059 | C | CT | 0.053 | ENST00000216037 | XBP1 | frameshift_variant |
| **MM183** | chr1:51436162 | CG | C | 0.919 | ENST00000371761 | CDKN2C | frameshift_variant |
| **MM183** | chr14:103371766 | G | T | 0.333 | ENST00000560371 | TRAF3 | missense_variant |
| **MM184** | chr12:25380275 | T | G | 0.283 | ENST00000311936 | KRAS | missense_variant |
| **MM184** | chr16:79633561 | G | T | 0.15 | ENST00000326043 | MAF | stop_gained |
| **MM185** | chr2:209106801 | G | A | 0.528 | ENST00000446179 | IDH1 | missense_variant |
| **MM185** | chr17:40474319 | A | T | 0.472 | ENST00000264657 | STAT3 | missense_variant |
| **MM185** | chr16:50785599 | GT | G | 0.024 | ENST00000311559 | CYLD | frameshift_variant |
| **MM185** | chr16:50785647 | G | T | 0.03 | ENST00000311559 | CYLD | stop_gained |
| **MM185** | chr16:50785792 | T | G | 0.287 | ENST00000311559 | CYLD | stop_gained |
| **MM186** | chr5:137801584 | C | T | 0.348 | ENST00000239938 | EGR1 | missense_variant |
| **MM186** | chr5:137801630 | C | A | 0.342 | ENST00000239938 | EGR1 | missense_variant |
| **MM186** | chr8:77754978 | G | T | 0.129 | ENST00000521891 | ZFHX4 | missense_variant |
| **MM187** | chr7:140453136 | A | T | 0.473 | ENST00000288602 | BRAF | missense_variant |
| **MM187** | chr22:23230235 | T | C | 0.351 | ENST00000526893 | IGLL5 | initiator_codon_variant |
| **MM187** | chr15:90631934 | C | T | 0.046 | ENST00000330062 | IDH2 | missense_variant |
| **MM187** | chr9:120476525 | C | T | 0.137 | ENST00000355622 | TLR4 | missense_variant |
| **MM188** | chr22:23230331 | T | A | 0.408 | ENST00000526893 | IGLL5 | missense_variant |
| **MM189** | chr11:108178709 | G | C | 0.294 | ENST00000278616 | ATM | splice_region_variant\|missens e_variant |

| **MM189** | chr12:25378561 | G | A | 0.411 | ENST00000311936 | KRAS | missense_variant |
| --- | --- | --- | --- | --- | --- | --- | --- |
| **MM189** | chr13:73349482 | C | T | 0.828 | ENST00000377767 | DIS3 | missense_variant |
| **MM189** | chr14:35871979 | G | A | 0.033 | ENST00000216797 | NFKBIA | splice_region_variant\|stop_ga ined |
| **MM189** | chr9:139814894 | TG | T | 0.862 | ENST00000247668 | TRAF2 | frameshift_variant |
| **MM190** | chr11:108178709 | G | C | 0.256 | ENST00000278616 | ATM | splice_region_variant\|missens e_variant |
| **MM190** | chr12:25378561 | G | A | 0.339 | ENST00000311936 | KRAS | missense_variant |
| **MM190** | chr13:73349482 | C | T | 0.462 | ENST00000377767 | DIS3 | missense_variant |
| **MM190** | chr17:7576855 | G | A | 0.447 | ENST00000269305 | TP53 | splice_region_variant\|stop_ga ined |
| **MM190** | chr9:139814894 | TG | T | 0.808 | ENST00000247668 | TRAF2 | frameshift_variant |
| **MM191** | chr1:115256528 | T | A | 0.055 | ENST00000369535 | NRAS | missense_variant |
| **MM191** | chrX:119663952 | C | T | 0.047 | ENST00000404115 | CUL4B | splice_donor_5th_base_varian t |
| **MM192** | chr22:23230348 | C | G | 0.472 | ENST00000526893 | IGLL5 | missense_variant |
| **MM192** | chr12:25398284 | C | T | 0.034 | ENST00000311936 | KRAS | missense_variant |
| **MM192** | chr11:108098354 | G | C | 0.045 | ENST00000278616 | ATM | initiator_codon_variant |
| **MM192** | chr11:108216612 | G | A | 0.426 | ENST00000278616 | ATM | missense_variant |
| **MM192** | chr17:37933933 | C | T | 0.027 | ENST00000346872 | IKZF3 | missense_variant |
| **MM192** | chr2:231118123 | G | A | 0.263 | ENST00000392045 | SP140 | missense_variant |
| **MM193** | chr7:50367287 | C | T | 0.021 | ENST00000331340 | IKZF1 | missense_variant |
| **MM193** | chr7:106509222 | G | C | 0.522 | ENST00000496166 | PIK3CG | missense_variant |
| **MM194** | chr7:140453136 | A | T | 0.461 | ENST00000288602 | BRAF | missense_variant |
| **MM194** | chr1:154941027 | T | C | 0.278 | ENST00000448116 | SHC1 | missense_variant |
| **MM194** | chr11:108114845 | G | A | 0.03 | ENST00000278616 | ATM | splice_region_variant\|missens e_variant |
| **MM194** | chr11:108117780 | A | T | 0.028 | ENST00000278616 | ATM | stop_gained |
| **MM194** | chr17:7578210 | TCGAAAAG | T | 0.038 | ENST00000269305 | TP53 | frameshift_variant |
| **MM194** | chr2:231162158 | A | G | 0.444 | ENST00000392045 | SP140 | missense_variant |
| **MM194** | chr2:231944886 | CA | C | 0.129 | ENST00000308696 | PSMD1 | frameshift_variant |
| **MM194** | chr2:231947621 | G | C | 0.016 | ENST00000308696 | PSMD1 | missense_variant |
| **MM194** | chr6:394912 | A | T | 0.491 | ENST00000380956 | IRF4 | missense_variant |
| **MM195** | chr4:1808659 | G | A | 0.488 | ENST00000440486 | FGFR3 | splice_region_variant\|missens e_variant |
| **MM195** | chr13:73349406 | T | TA | 0.204 | ENST00000377767 | DIS3 | frameshift_variant |
| **MM195** | chr15:99250961 | C | T | 0.341 | ENST00000268035 | IGF1R | stop_gained |
| **MM195** | chr17:73322014 | A | T | 0.376 | ENST00000316804 | GRB2 | missense_variant |

| **MM196** | chr22:23230415 | C |  | G | 0.935 | ENST00000526893 | IGLL5 | missense_variant |
| --- | --- | --- | --- | --- | --- | --- | --- | --- |
| **MM196** | chr3:142272762 | T |  | C | 0.476 | ENST00000350721 | ATR | missense_variant |
| **MM196** | chr2:231101933 | G |  | A | 0.358 | ENST00000392045 | SP140 | missense_variant |
| **MM196** | chr22:23230349 | T |  | A | 0.962 | ENST00000526893 | IGLL5 | missense_variant |
| **MM196** | chr4:1803716 | CCGGACGGCA |  | G | 0.603 | ENST00000440486 | FGFR3 | frameshift_variant |
| **MM197** | chr15:90631934 | C |  | T | 0.049 | ENST00000330062 | IDH2 | missense_variant |
| **MM197** | chr1:118166208 | A |  | T | 0.01 | ENST00000369448 | FAM46C | stop_gained |
| **MM197** | chr11:108216576 | C |  | T | 0.876 | ENST00000278616 | ATM | missense_variant |
| **MM197** | chrX:119693958 | A |  | C | 0.091 | ENST00000404115 | CUL4B | stop_gained |
| **MM198** | chr8:77761888 | A |  | T | 0.531 | ENST00000521891 | ZFHX4 | missense_variant |
| **MM198** | chr12:25398284 | C |  | T | 0.369 | ENST00000311936 | KRAS | missense_variant |
| **MM198** | chr11:108186625 | C |  | A | 0.474 | ENST00000278616 | ATM | missense_variant |
| **MM198** | chr13:73346351 | A |  | C | 0.642 | ENST00000377767 | DIS3 | missense_variant |
| **MM198** | chr17:79479307 | T |  | C | 0.411 | ENST00000331925 | ACTG1 | missense_variant |
| **MM198** | chr7:106508424 | C |  | G | 0.551 | ENST00000496166 | PIK3CG | missense_variant |
| **MM199** | chr11:108213988 | T |  | G | 0.375 | ENST00000278616 | ATM | missense_variant |
| **MM199** | chr22:23230297 | C |  | T | 0.358 | ENST00000526893 | IGLL5 | stop_gained |
| **MM199** | chr11:108114752 | T |  | G | 0.368 | ENST00000278616 | ATM | missense_variant |
| **MM199** | chr12:25380275 | T |  | G | 0.253 | ENST00000311936 | KRAS | missense_variant |
| **MM199** | chr22:23230258 | G |  | T | 0.388 | ENST00000526893 | IGLL5 | missense_variant |
| **MM200** | chr12:25378647 | T |  | G | 0.034 | ENST00000311936 | KRAS | missense_variant |
| **MM200** | chr11:69456206 | T |  | A | 0.524 | ENST00000227507 | CCND1 | missense_variant |
| **MM200** | chr12:25398255 | G |  | T | 0.111 | ENST00000311936 | KRAS | missense_variant |
| **MM200** | chr16:50783880 | G |  | T | 0.281 | ENST00000311559 | CYLD | stop_gained |
| **MM200** | chr7:140453153 | A |  | T | 0.036 | ENST00000288602 | BRAF | missense_variant |
| **MM201** | chr12:25380275 | T |  | A | 0.251 | ENST00000311936 | KRAS | missense_variant |
| **MM201** | chr12:58145457 | C |  | T | 0.279 | ENST00000257904 | CDK4 | missense_variant |
| **MM201** | chr15:99251043 | C |  | A | 0.014 | ENST00000268035 | IGF1R | missense_variant |
| **MM202** | chr11:108188160 | G |  | A | 0.234 | ENST00000278616 | ATM | missense_variant |
| **MM202** | chr12:25380275 | T |  | G | 0.214 | ENST00000311936 | KRAS | missense_variant |
| **MM202** | chr12:25380276 | T |  | C | 0.076 | ENST00000311936 | KRAS | missense_variant |
| **MM202** | chr7:55249106 | G |  | A | 0.119 | ENST00000275493 | EGFR | missense_variant |
| **MM203** | chr7:140453136 | A |  | T | 0.424 | ENST00000288602 | BRAF | missense_variant |
| **MM203** | chr15:99456337 | G |  | A | 0.491 | ENST00000268035 | IGF1R | missense_variant |

| **MM204** | chr1:115256529 | T | A | 0.469 | ENST00000369535 | NRAS | missense_variant |
| --- | --- | --- | --- | --- | --- | --- | --- |
| **MM205** | chr17:7578503 | C | T | 0.227 | ENST00000269305 | TP53 | missense_variant |
| **MM206** | chr19:40742253 | T | C | 0.541 | ENST00000392038 | AKT2 | missense_variant |
| **MM206** | chr12:25398284 | C | T | 0.112 | ENST00000311936 | KRAS | missense_variant |
| **MM206** | chr2:231176276 | G | A | 0.918 | ENST00000392045 | SP140 | missense_variant |
| **MM206** | chr1:115256528 | T | A | 0.028 | ENST00000369535 | NRAS | missense_variant |
| **MM206** | chr1:118165758 | G | A | 0.054 | ENST00000369448 | FAM46C | missense_variant |
| **MM206** | chr12:25380268 | A | T | 0.012 | ENST00000311936 | KRAS | missense_variant |
| **MM206** | chr12:25380282 | G | C | 0.038 | ENST00000311936 | KRAS | missense_variant |
| **MM206** | chr7:140477861 | T | C | 0.055 | ENST00000288602 | BRAF | missense_variant |
| **MM207** | chr1:115256530 | G | T | 0.069 | ENST00000369535 | NRAS | missense_variant |
| **MM207** | chr1:118165957 | C | CCCTCT | 0.116 | ENST00000369448 | FAM46C | frameshift_variant |
| **MM207** | chr14:65560458 | G | A | 0.015 | ENST00000358664 | MAX | missense_variant |
| **MM207** | chr22:29196385 | T | G | 0.522 | ENST00000216037 | XBP1 | missense_variant |
| **MM207** | chr7:140481417 | C | A | 0.117 | ENST00000288602 | BRAF | missense_variant |
| **MM207** | chrX:119677621 | T | A | 0.095 | ENST00000404115 | CUL4B | missense_variant |
| **MM208** | chr12:25380276 | T | C | 0.486 | ENST00000311936 | KRAS | missense_variant |
| **MM208** | chr6:394972 | A | G | 0.461 | ENST00000380956 | IRF4 | missense_variant |
| **MM209** | chr12:25380275 | T | G | 0.376 | ENST00000311936 | KRAS | missense_variant |
| **MM209** | chr2:231936988 | A | G | 0.017 | ENST00000308696 | PSMD1 | missense_variant |
| **MM210** | chr1:115256529 | T | A | 0.554 | ENST00000369535 | NRAS | missense_variant |
| **MM210** | chr1:118166301 | T | A | 0.798 | ENST00000369448 | FAM46C | missense_variant |
| **MM210** | chr13:73335930 | G | A | 0.946 | ENST00000377767 | DIS3 | missense_variant |
| **MM210** | chr8:77617630 | C | T | 0.47 | ENST00000521891 | ZFHX4 | missense_variant |
| **MM211** | chr11:108124558 | A | T | 0.477 | ENST00000278616 | ATM | missense_variant |
| **MM211** | chr13:73355010 | G | C | 0.922 | ENST00000377767 | DIS3 | missense_variant |
| **MM211** | chr7:55240719 | C | T | 0.51 | ENST00000275493 | EGFR | missense_variant |
| **MM212** | chr1:115256529 | T | A | 0.409 | ENST00000369535 | NRAS | missense_variant |
| **MM212** | chr11:108168047 | T | C | 0.475 | ENST00000278616 | ATM | missense_variant |
| **MM213** | chr13:73351570 | CAA | C | 0.925 | ENST00000377767 | DIS3 | inframe |
| **MM213** | chr17:7578403 | C | T | 0.972 | ENST00000269305 | TP53 | missense_variant |
| **MM213** | chr17:40475068 | G | T | 0.453 | ENST00000264657 | STAT3 | missense_variant |
| **MM213** | chr4:1808957 | GTGAAGGGCC | G | 0.929 | ENST00000440486 | FGFR3 | stop_lost |
| **MM213** | chr9:120475394 | T | C | 0.013 | ENST00000355622 | TLR4 | missense_variant |

| **MM214** | chr2:231102962 | T | G | 0.414 | ENST00000392045 | SP140 | missense_variant |
| --- | --- | --- | --- | --- | --- | --- | --- |
| **MM214** | chr4:15818245 | G | C | 0.078 | ENST00000226279 | CD38 | missense_variant |
| **MM215** | chr1:115256529 | T | C | 0.598 | ENST00000369535 | NRAS | missense_variant |
| **MM215** | chr13:73336145 | T | C | 0.816 | ENST00000377767 | DIS3 | missense_variant |
| **MM215** | chr3:30713210 | G | A | 0.524 | ENST00000295754 | TGFBR2 | missense_variant |
| **MM215** | chrX:119691790 | C | A | 0.057 | ENST00000404115 | CUL4B | stop_gained |
| **MM216** | chr1:1647878 | C | T | 0.038 | ENST00000404249 | CDK11A | missense_variant |
| **MM216** | chr22:23230403 | G | C | 0.636 | ENST00000526893 | IGLL5 | missense_variant |
| **MM217** | chr2:96810701 | C | T | 0.455 | ENST00000288943 | DUSP2 | splice_donor_5th_base_varian t |
| **MM217** | chr7:140453136 | A | T | 0.057 | ENST00000288602 | BRAF | missense_variant |
| **MM217** | chr1:115256528 | T | G | 0.142 | ENST00000369535 | NRAS | missense_variant |
| **MM217** | chr1:115256529 | T | C | 0.029 | ENST00000369535 | NRAS | missense_variant |
| **MM217** | chr1:115256530 | G | T | 0.058 | ENST00000369535 | NRAS | missense_variant |
| **MM217** | chr1:118166309 | G | C | 0.072 | ENST00000369448 | FAM46C | missense_variant |
| **MM218** | chr11:108139232 | C | T | 0.444 | ENST00000278616 | ATM | stop_gained |
| **MM218** | chr12:25398284 | C | G | 0.49 | ENST00000311936 | KRAS | missense_variant |
| **MM219** | chr12:25398284 | C | A | 0.415 | ENST00000311936 | KRAS | missense_variant |
| **MM220** | chr1:115258744 | C | T | 0.46 | ENST00000369535 | NRAS | missense_variant |
| **MM220** | chr1:152187203 | C | G | 0.104 | ENST00000368801 | HRNR | missense_variant |
| **MM220** | chr5:142779605 | C | T | 0.031 | ENST00000343796 | NR3C1 | missense_variant |
| **MM220** | chr6:394972 | A | G | 0.531 | ENST00000380956 | IRF4 | missense_variant |
| **MM221** | chr1:115256529 | T | C | 0.047 | ENST00000369535 | NRAS | missense_variant |
| **MM222** | chr13:73349406 | T | TA | 0.299 | ENST00000377767 | DIS3 | frameshift_variant |
| **MM222** | chr15:99250961 | C | T | 0.333 | ENST00000268035 | IGF1R | stop_gained |
| **MM222** | chr17:73322014 | A | T | 0.195 | ENST00000316804 | GRB2 | missense_variant |
| **MM223** | chr10:104160239 | G | T | 0.333 | ENST00000369966 | NFKB2 | missense_variant |
| **MM223** | chr12:25398255 | G | T | 0.441 | ENST00000311936 | KRAS | missense_variant |
| **MM223** | chr3:178921426 | C | A | 0.179 | ENST00000263967 | PIK3CA | missense_variant |
| **MM223** | chr6:106554331 | A | G | 0.019 | ENST00000369096 | PRDM1 | missense_variant |
| **MM224** | chr12:25398284 | C | T | 0.011 | ENST00000311936 | KRAS | missense_variant |
| **MM224** | chr1:51439661 | G | A | 0.589 | ENST00000371761 | CDKN2C | missense_variant |
| **MM224** | chr1:115256528 | T | G | 0.237 | ENST00000369535 | NRAS | missense_variant |
| **MM224** | chr12:25380275 | T | G | 0.289 | ENST00000311936 | KRAS | missense_variant |
| **MM224** | chr2:136873382 | A | G | 0.481 | ENST00000241393 | CXCR4 | missense_variant |

| **MM225** | chr12:12870955 | ATTTCGAT |  | A | 0.83 | ENST00000228872 | CDKN1B | frameshift_variant |
| --- | --- | --- | --- | --- | --- | --- | --- | --- |
| **MM225** | chr8:77617335 | C |  | T | 0.622 | ENST00000521891 | ZFHX4 | missense_variant |
| **MM226** | chr1:115256530 | G |  | T | 0.296 | ENST00000369535 | NRAS | missense_variant |
| **MM227** | chr17:7577075 | T |  | TTC | 0.612 | ENST00000269305 | TP53 | frameshift_variant |
| **MM227** | chr17:37947690 | C |  | T | 0.428 | ENST00000346872 | IKZF3 | missense_variant |
| **MM228** | chr3:141289864 | A |  | G | 0.574 | ENST00000452898 | RASA2 | missense_variant |
| **MM229** | chr12:25380276 | T |  | A | 0.555 | ENST00000311936 | KRAS | missense_variant |
| **MM229** | chr13:48936978 | AACACCCAGG |  | C | 0.629 | ENST00000267163 | RB1 | frameshift_variant |
| **MM229** | chr17:37922170 | G |  | A | 0.443 | ENST00000346872 | IKZF3 | missense_variant |
| **MM229** | chr8:77764579 | C |  | A | 0.43 | ENST00000521891 | ZFHX4 | missense_variant |
| **MM230** | chr12:25398284 | C |  | T | 0.406 | ENST00000311936 | KRAS | missense_variant |
| **MM230** | chr12:25378562 | C |  | T | 0.464 | ENST00000311936 | KRAS | missense_variant |
| **MM230** | chr4:106157753 | ACAGGAGCA |  | A | 0.8 | ENST00000380013 | TET2 | frameshift_variant |
| **MM231** | chr14:103363633 | CAA |  | C | 0.245 | ENST00000560371 | TRAF3 | frameshift_variant |
| **MM231** | chr5:137801478 | C |  | G | 0.479 | ENST00000239938 | EGR1 | missense_variant |
| **MM232** | chr1:118166268 | C |  | T | 0.225 | ENST00000369448 | FAM46C | stop_gained |
| **MM232** | chr13:73355001 | G |  | C | 0.026 | ENST00000377767 | DIS3 | missense_variant |
| **MM233** | chr11:108188160 | G |  | A | 0.164 | ENST00000278616 | ATM | missense_variant |
| **MM233** | chr12:25380275 | T |  | G | 0.289 | ENST00000311936 | KRAS | missense_variant |
| **MM233** | chr7:55249106 | G |  | A | 0.048 | ENST00000275493 | EGFR | missense_variant |
| **MM234** | chr22:23230406 | G |  | T | 0.38 | ENST00000526893 | IGLL5 | missense_variant |
| **MM235** | chr22:29195088 | C |  | T | 0.036 | ENST00000216037 | XBP1 | missense_variant |
| **MM235** | chr3:30729991 | G |  | A | 0.082 | ENST00000295754 | TGFBR2 | stop_gained |
| **MM236** | chr1:118166556 | T |  | G | 0.196 | ENST00000369448 | FAM46C | missense_variant |
| **MM236** | chr12:25380276 | T |  | C | 0.24 | ENST00000311936 | KRAS | missense_variant |
| **MM236** | chr6:3113361 | G |  | A | 0.217 | ENST00000380409 | RIPK1 | missense_variant |
| **MM237** | chr12:25380275 | T |  | A | 0.189 | ENST00000311936 | KRAS | missense_variant |
| **MM238** | chr11:108153564 | CTTTTA |  | C | 0.59 | ENST00000278616 | ATM | frameshift_variant |
| **MM238** | chr17:7577506 | C |  | G | 0.544 | ENST00000269305 | TP53 | missense_variant |
| **MM238** | chr2:231941798 | GAGTTACATCT |  | T | 0.22 | ENST00000308696 | PSMD1 | frameshift_variant |
| **MM238** | chr2:231944327 | A |  | T | 0.038 | ENST00000308696 | PSMD1 | splice_region_variant\|missens e_variant |
| **MM238** | chr3:3197922 | A |  | AGC | 0.778 | ENST00000231948 | CRBN | frameshift_variant |
| **MM238** | chr5:67576802 | C |  | G | 0.478 | ENST00000521381 | PIK3R1 | stop_gained |
| **MM238** | chr8:77616838 | C |  | T | 0.071 | ENST00000521891 | ZFHX4 | missense_variant |

| **MM238** | chr9:21971068 | A | T | 0.529 | ENST00000304494 | CDKN2A | missense_variant |
| --- | --- | --- | --- | --- | --- | --- | --- |
| **MM238** | chr9:120475372 | G | T | 0.1 | ENST00000355622 | TLR4 | missense_variant |
| **MM239** | chr17:73317767 | G | T | 0.026 | ENST00000316804 | GRB2 | missense_variant |
| **MM239** | chr5:68572506 | C | A | 0.02 | ENST00000256443 | CDK7 | missense_variant |
| **MM240** | chr10:104157770 | C | T | 0.357 | ENST00000369966 | NFKB2 | stop_gained |
| **MM240** | chr12:25380275 | T | G | 0.035 | ENST00000311936 | KRAS | missense_variant |
| **MM241** | chr1:115256529 | T | C | 0.043 | ENST00000369535 | NRAS | missense_variant |
| **MM241** | chr11:108236060 | A | T | 0.502 | ENST00000278616 | ATM | missense_variant |
| **MM241** | chr12:92539194 | A | G | 0.32 | ENST00000256015 | BTG1 | missense_variant |
| **MM241** | chr12:92539203 | G | T | 0.298 | ENST00000256015 | BTG1 | missense_variant |
| **MM241** | chr2:231162182 | G | A | 0.043 | ENST00000392045 | SP140 | splice_donor_5th_base_varian t |
| **MM241** | chr5:55259291 | C | G | 0.015 | ENST00000381298 | IL6ST | missense_variant |
| **MM243** | chr12:25380275 | T | G | 0.441 | ENST00000311936 | KRAS | missense_variant |
| **MM243** | chr17:7578466 | G | T | 1 | ENST00000269305 | TP53 | missense_variant |
| **MM244** | chr17:7578403 | C | A | 0.94 | ENST00000269305 | TP53 | missense_variant |
| **MM244** | chr3:142274917 | C | G | 0.447 | ENST00000350721 | ATR | missense_variant |
| **MM244** | chr5:67591110 | C | G | 0.373 | ENST00000521381 | PIK3R1 | missense_variant |
| **MM244** | chr5:142780340 | T | C | 0.507 | ENST00000343796 | NR3C1 | missense_variant |
| **MM242** | chr1:118165587 | G | GT | 0.115 | ENST00000369448 | FAM46C | frameshift_variant |
| **MM242** | chr14:103338280 | G | T | 0.117 | ENST00000560371 | TRAF3 | missense_variant |
| **MM242** | chr2:231120181 | T | C | 0.418 | ENST00000392045 | SP140 | missense_variant |
| **MM245** | chr12:25380275 | T | G | 0.342 | ENST00000256078 | KRAS | missense_variant |
| **MM246** | chr13:73346329 | G | A | 0.027 | ENST00000377767 | DIS3 | missense_variant |
| **MM246** | chr13:73355901 | A | T | 0.042 | ENST00000377767 | DIS3 | missense_variant |
| **MM246** | chr13:73355087 | G | A | 0.09 | ENST00000377767 | DIS3 | missense_variant |
| **MM246** | chr13:73345240 | G | A | 0.457 | ENST00000377767 | DIS3 | missense_variant |
| **MM246** | chr12:25398284 | C | T | 0.203 | ENST00000256078 | KRAS | missense_variant |
| **MM246** | chr1:115256530 | G | T | 0.023 | ENST00000369535 | NRAS | missense_variant |
| **MM246** | chr9:139802527 | C | A | 0.036 | ENST00000247668 | TRAF2 | stop_gained |
| **MM246** | chr7:140453193 | T | G | 0.5 | ENST00000288602 | BRAF | splice_region_variant\|missens e_variant |
| **MM246** | chr1:115256528 | T | G | 0.026 | ENST00000369535 | NRAS | missense_variant |
| **MM247** | chr2:96810841 | G | C | 0.5 | ENST00000288943 | DUSP2 | missense_variant |
| **MM248** | chr12:25378647 | T | G | 0.38 | ENST00000256078 | KRAS | missense_variant |
| **MM249** | chr8:77617450 | C | T | 0.095 | ENST00000521891 | ZFHX4 | missense_variant |

| **MM249** | chr7:140453136 | A | T | 0.103 | ENST00000288602 | BRAF | missense_variant |
| --- | --- | --- | --- | --- | --- | --- | --- |
| **MM250** | chr5:137801688 | A | T | 0.466 | ENST00000239938 | EGR1 | missense_variant |
| **MM250** | chr4:1808988 | G | T | 0.433 | ENST00000340107 | FGFR3 | stop_lost |
| **MM251** | chr14:65543280 | C | T | 0.495 | ENST00000358664 | MAX | missense_variant |
| **MM251** | chr1:115258744 | C | T | 0.063 | ENST00000369535 | NRAS | missense_variant |
| **MM251** | chr17:40990120 | G | GTTTGTGA | 0.082 | ENST00000293362 | PSME3 | frameshift_variant |
| **MM252** | chr11:69457938 | T | A | 0.345 | ENST00000227507 | CCND1 | missense_variant |
| **MM252** | chr13:73334788 | A | G | 0.468 | ENST00000377767 | DIS3 | splice_region_variant\|missens e_variant |
| **MM252** | chr1:118165803 | T | G | 0.629 | ENST00000369448 | FAM46C | missense_variant |
| **MM253** | chr12:25380275 | T | G | 0.612 | ENST00000256078 | KRAS | missense_variant |
| **MM254** | chr7:106513269 | G | A | 0.263 | ENST00000359195 | PIK3CG | missense_variant |
| **MM255** |  |  |  |  |  |  |  |
| **MM256** | chrX:44918571 | G | A | 1 | ENST00000377967 | KDM6A | missense_variant |
| **MM256** | chr11:108199946 | C | G | 0.663 | ENST00000278616 | ATM | missense_variant |
| **MM257** | chr13:73355117 | T | G | 0.412 | ENST00000377767 | DIS3 | missense_variant |
| **MM257** | chr12:25380275 | T | G | 0.244 | ENST00000256078 | KRAS | missense_variant |
| **MM257** | chr6:106547259 | CA | C | 0.011 | ENST00000369096 | PRDM1 | frameshift_variant |
| **MM257** | chr17:40475064 | C | T | 0.017 | ENST00000264657 | STAT3 | missense_variant |
| **MM258** | chr1:115256529 | T | C | 0.319 | ENST00000369535 | NRAS | missense_variant |
| **MM259** | chr17:65337018 | G | C | 0.423 | ENST00000356126 | PSMD12 | missense_variant |
| **MM259** | chr17:7578445 | A | C | 0.422 | ENST00000269305 | TP53 | missense_variant |
| **MM259** | chr22:39917509 | T | TTG | 0.496 | ENST00000337304 | ATF4 | frameshift_variant |
| **MM259** | chr1:154420627 | G | A | 0.464 | ENST00000368485 | IL6R | missense_variant |
| **MM259** | chr4:1902658 | G | A | 0.482 | ENST00000382895 | WHSC1 | missense_variant |
| **MM260** | chr7:55224270 | A | G | 0.357 | ENST00000275493 | EGFR | missense_variant |
| **MM260** | chrX:44929485 | C | T | 0.2 | ENST00000377967 | KDM6A | missense_variant |
| **MM260** | chr12:25378647 | T | A | 0.164 | ENST00000256078 | KRAS | missense_variant |
| **MM260** | chr2:88926720 | C | T | 0.41 | ENST00000303236 | EIF2AK3 | missense_variant |
| **MM260** | chr3:141305596 | T | A | 0.253 | ENST00000286364 | RASA2 | splice_donor_variant |
| **MM261** | chr6:393348 | G | A | 0.363 | ENST00000380956 | IRF4 | missense_variant |
| **MM261** | chr10:104156749 | G | A | 0.015 | ENST00000369966 | NFKB2 | missense_variant |
| **MM261** | chr12:49088050 | GGAAGGCAC | G | 0.26 | ENST00000261900 | CCNT1 | frameshift_variant |
| **MM261** | chr1:115256528 | T | G | 0.431 | ENST00000369535 | NRAS | missense_variant |
| **MM262** | chr2:231177366 | GT | G | 0.5 | ENST00000392045 | SP140 | frameshift_variant |

| **MM262** | chr12:25398284 | C | T | 0.086 | ENST00000256078 | KRAS | missense_variant |
| --- | --- | --- | --- | --- | --- | --- | --- |
| **MM262** | chr12:25398285 | C | G | 0.237 | ENST00000256078 | KRAS | missense_variant |
| **MM263** | chr1:115256521 | A | C | 0.69 | ENST00000369535 | NRAS | missense_variant |
| **MM264** | chr22:29196436 | G | A | 0.596 | ENST00000216037 | XBP1 | missense_variant |
| **MM264** | chr22:29195089 | G | A | 0.474 | ENST00000216037 | XBP1 | stop_gained |
| **MM264** | chr22:29195088 | C | T | 0.502 | ENST00000216037 | XBP1 | missense_variant |
| **MM264** | chr11:108155010 | T | G | 0.297 | ENST00000278616 | ATM | missense_variant |
| **MM264** | chr3:142254029 | C | T | 0.331 | ENST00000350721 | ATR | missense_variant |
| **MM264** | chr3:3195156 | C | T | 0.294 | ENST00000231948 | CRBN | missense_variant |
| **MM264** | chr3:3221362 | C | T | 0.483 | ENST00000231948 | CRBN | missense_variant |
| **MM264** | chr5:137801740 | A | C | 0.492 | ENST00000239938 | EGR1 | missense_variant |
| **MM264** | chr12:25398284 | C | T | 0.628 | ENST00000256078 | KRAS | missense_variant |
| **MM264** | chr2:231152642 | C | T | 0.396 | ENST00000392045 | SP140 | stop_gained |
| **MM264** | chr17:7576855 | G | A | 0.466 | ENST00000269305 | TP53 | splice_region_variant\|stop_ga ined |
| **MM265** | chr1:243809211 | G | C | 0.367 | ENST00000366539 | AKT3 | missense_variant |
| **MM265** | chr1:115256529 | T | C | 0.404 | ENST00000369535 | NRAS | missense_variant |
| **MM265** | chr6:394972 | A | G | 0.07 | ENST00000380956 | IRF4 | missense_variant |
| **MM265** | chr17:30796031 | C | T | 0.471 | ENST00000261712 | PSMD11 | missense_variant |

**Supplementary Table 3: Sensitivity, specificity, positive predictive value (PPV), and negative predictive value (NPV) in the detection of rearrangements and copy number variations (CNV) by FISH and NGS.**

|  | **Tri3** | **Tri7** | **Tri9** | **Tri11** | **Tri15** | **t(11;14)** | **t(4;14)** | **t(14;16)** | **t(6;14)** | **t(14;20)** | **Mono(13)** | **Gain(1q)** | **Del(17p)** |
| --- | --- | --- | --- | --- | --- | --- | --- | --- | --- | --- | --- | --- | --- |
| **Sensitivity** | 90.30% | 92.70% | 92.50% | 94.20% | 95% | 97% | 100% | 100% | 100% | 100% | 97% | 94.60% | 78% |
| **Specificity** | 93.30% | 87.40% | 79.60% | 87% | 77.20% | 100% | 100% | 100% | 100% | 100% | 95% | 91.40% | 100% |
| **PPV** | 90.30% | 80.90% | 83.50% | 81.80% | 76.80% | 100% | 100% | 100% | 100% | 100% | 94.20% | 91.40% | 100% |
| **NPV** | 93.30% | 95.40% | 90.50% | 96% | 95.10% | 98.80% | 100% | 100% | 100% | 100% | 97.40% | 94.60% | 97.5% |

**Supplementary Table 4: Patients in the cohort with the presence of *TP53* mutation, deletion of chromosome 17p (del(17p)), and/or loss of heterozygosity (LOH). Disease stage categories include MGUS, SMM, newly diagnosed multiple myeloma (NDMM) and relapsed/refractory multiple myeloma (RRMM).**

| **Disease stage** | ***TP53* Mutation** | **17p del** | **LOH** |
| --- | --- | --- | --- |
| MGUS | X |  |  |
| NDMM | X | X |  |
| NDMM | X | X |  |
| NDMM |  | X |  |
| NDMM | X | X |  |
| NDMM | X | X |  |
| NDMM | X | X |  |
| NDMM | X | X | X |
| NDMM |  | X |  |
| NDMM | X |  |  |
| NDMM | X |  |  |
| NDMM | X |  |  |
| NDMM | X |  |  |
| NDMM | X |  | X |
| NDMM | X |  |  |
| NDMM | X | X |  |
| NDMM | X |  |  |
| NDMM | X | X |  |
| NDMM |  | X |  |
| NDMM | X |  |  |
| NDMM |  | X |  |
| NDMM |  | X |  |
| NDMM |  | X |  |
| NDMM | X | X |  |
| NDMM | X | X |  |
| NDMM |  | X |  |
| NDMM | X |  |  |
| NDMM | X |  |  |
| RRMM |  | X |  |
| RRMM | X | X |  |
| RRMM | X |  |  |
| RRMM | X |  |  |
| RRMM |  | X |  |
| RRMM |  | X |  |
| RRMM | X | X | X |
| RRMM | X |  |  |
| RRMM |  | X |  |
| RRMM | X |  |  |
| RRMM |  | X |  |
| RRMM |  | X |  |
| RRMM | X | X |  |
| RRMM | X |  |  |
| RRMM | X |  |  |
| RRMM | X | X | X |
